# A LOV Protein Modulates the Physiological Attributes of *Xanthomonas axonopodis* pv. citri Relevant for Host Plant Colonization

**DOI:** 10.1371/journal.pone.0038226

**Published:** 2012-06-04

**Authors:** Ivana Kraiselburd, Analía I. Alet, María Laura Tondo, Silvana Petrocelli, Lucas D. Daurelio, Jesica Monzón, Oscar A. Ruiz, Aba Losi, Elena G. Orellano

**Affiliations:** 1 Molecular Biology Division, Instituto de Biología Molecular y Celular de Rosario (IBR), CONICET, Facultad de Ciencias Bioquímicas y Farmacéuticas, Universidad Nacional de Rosario, Rosario, Argentina; 2 IIB-INTECH, Unidad de Biotecnología, Chascomús, Buenos Aires, Argentina; 3 Department of Physics, University of Parma, Parma, Italy; Universidad de Sevilla, Spain

## Abstract

Recent studies have demonstrated that an appropriate light environment is required for the establishment of efficient vegetal resistance responses in several plant-pathogen interactions. The photoreceptors implicated in such responses are mainly those belonging to the phytochrome family. Data obtained from bacterial genome sequences revealed the presence of photosensory proteins of the BLUF (Blue Light sensing Using FAD), LOV (Light, Oxygen, Voltage) and phytochrome families with no known functions. *Xanthomonas axonopodis* pv. citri is a Gram-negative bacterium responsible for citrus canker. The *in silico* analysis of the *X. axonopodis* pv. citri genome sequence revealed the presence of a gene encoding a putative LOV photoreceptor, in addition to two genes encoding BLUF proteins. This suggests that blue light sensing could play a role in *X. axonopodis* pv. citri physiology. We obtained the recombinant Xac-LOV protein by expression in *Escherichia coli* and performed a spectroscopic analysis of the purified protein, which demonstrated that it has a canonical LOV photochemistry. We also constructed a mutant strain of *X. axonopodis* pv. citri lacking the LOV protein and found that the loss of this protein altered bacterial motility, exopolysaccharide production and biofilm formation. Moreover, we observed that the adhesion of the mutant strain to abiotic and biotic surfaces was significantly diminished compared to the wild-type. Finally, inoculation of orange (*Citrus sinensis*) leaves with the mutant strain of *X. axonopodis* pv. citri resulted in marked differences in the development of symptoms in plant tissues relative to the wild-type, suggesting a role for the Xac-LOV protein in the pathogenic process. Altogether, these results suggest the novel involvement of a photosensory system in the regulation of physiological attributes of a phytopathogenic bacterium. A functional blue light receptor in *Xanthomonas* spp. has been described for the first time, showing an important role in virulence during citrus canker disease.

## Introduction

Light is a major environmental stimulus that regulates plant physiology. Among light-regulated vegetal responses are those elicited by the attack of pathogens, and the requirement of an adequate light environment for a full defense response has been extensively studied [1]–[3]. The perception of light has also been linked to numerous physiological responses in microorganisms such as pigment synthesis, DNA repair and biofilm formation [4]. Moreover, recent reports revealed that in many bacteria light governs important lifestyle decisions, especially that between a single-cell motile state and a multicellular surface-attached state [5]. Furthermore, the participation of light in the regulation of bacterial virulence was recently found in non-photosynthetic bacteria. The presence of light receptors across several bacterial taxa, including many species with no known photobiology, suggests that the visible light environment has an unexplored regulatory role in the biology of bacterial cells [4], [6]–[8].

The perception of light in both eukaryotic and prokaryotic organisms is conducted via photoreceptor proteins that belong to one of six families defined by the structure of their light-absorbing molecules or chromophores [9]. Among blue light receptors are proteins with LOV (Light, Oxygen or Voltage) and BLUF (Blue Light sensing Using FAD) domains. LOV domains are small photosensing protein modules (around 100 amino acids) that constitute a subclass of the widespread PAS (Per-Arnt-Sim) superfamily [10]. Several PAS-domain proteins are known to detect environmental signals by way of an associated cofactor [11], as is the case with LOV proteins. The best-characterized LOV proteins are plant phototropins, photoreceptors involved in phototropic bending, light-induced stomatal opening and light-directed chloroplast movement [12]. LOV domains contain a molecule of flavin mononucleotide (FMN) as a non-covalently bound chromophore. For this reason, they maximally absorb light near 450 nm, and show a strong fluorescence emission at 500 nm upon the excitation of the flavin. The photochemistry of LOV domains was first elucidated for phototropins [13] and afterward for a variety of bacterial and fungal proteins [14], [15]. This photochemistry involves the formation of a photoadduct (by the creation of a covalent bond between the carbon atom at position 4a of FMN and the thiol group of a conserved cysteine located in the LOV domain) that is significantly blue-shifted with respect to the dark state and it is non-fluorescent [7], [16], [17].

Data obtained from bacterial genome sequences revealed the presence of blue light photosensory proteins belonging to the BLUF and LOV families in many prokaryotic species [4], [6]. Losi and Gärtner found 307 proteins containing LOV domains in the genomes of 227 bacterial species [14]. Despite the large number of bacterial photoreceptors found to date, the physiological implications of these proteins are poorly understood. A few reports have been presented for the physiological function of blue light photoreceptors in important pathogens, e.g., *Brucella abortus* [7], *Acinetobacter baumanni* [18], *Escherichia coli* [19] and *Listeria monocytogenes* [20]. In non-pathogenic bacteria, photoresponses linked to LOV and BLUF proteins have been reported for *Bacillus subtilis* [21], [22], *Caulobacter crescentus* [23] and *Rhodopseudomonas palustris* [14], [24]. Regarding phytopathogens, the presence of different blue light receptors including LOV and BLUF proteins, have been reported for several microorganisms such as *Xanthomonas*, *Pseudomonas* and *Ralstonia* [4], [25]; however, little is known about the role of these proteins on bacterial physiology. This topic has been recently discussed in a dedicated review [26]. In addition, a LOV protein from *Pseudomonas syringae* pv. tomato was confirmed as a blue light-regulated kinase with a still undetermined physiological role [7], [27], and the light-regulated effects over *Agrobacterium tumefaciens* were described [28].

*Xanthomonas axonopodis* pv. citri is a Gram-negative bacterium responsible for citrus canker, a severe disease that affects all citrus cultivars [29], [30]. The pathogen enters host plant tissues through stomata and wounds. Subsequently, bacteria colonize the apoplast causing the leaf epidermis to break due to cell hyperplasia. The disease ultimately appears as raised necrotic corky lesions on leaves, stems and fruit surfaces, reducing the fruit quality and quantity [29], [30]. The *X. axonopodis* pv. citri genome contains three genes encoding putative blue light photoreceptors: two BLUF proteins and one LOV protein. The LOV protein (Xac-LOV) is encoded by the *fixL* gene, which was renamed as *lov* gene for clarity purposes [31], [32]. In this work, we studied the potential blue light activation of the LOV protein present in this phytopathogen by evaluating blue light-induced spectral changes of the purified recombinant protein. We also investigated the influence of blue light and the LOV protein on the physiology and infectivity of *X.* *axonopodis* pv. citri. To this aim we constructed a mutant strain lacking a functional *lov* gene and studied the effect of the absence of this gene on bacterial physiological features and on the interaction between *X. axonopodis* pv. citri and its host plants.

## Results

### Xac-LOV is a Typical Histidine Kinase-response Regulator Hybrid Protein

The first evidence for the occurrence of LOV proteins in bacteria was presented by Huala *et al*. [33], who compared the sequences of phototropin-LOV domains with those present in the LOV proteins of *B. subtilis* (gene *ytvA*) and *Synechocystis* PCC 6803 Q55576 (gene *slr0359*). In addition, Crosson *et al.* reported a sequence alignment including additional bacterial LOV proteins from *X. axonopodis* pv. citri, *Xanthomonas campestris*, *C. crescentus*, *Brucella melitensis*, *Nostoc* sp. PCC 7120, *Listeria monocytogenes* and *Listeria innocua* [8], [34]. In *X. axonopodis* pv. citri, the LOV protein is encoded by the *fixL* gene (accession number AAM37406.1), which we renamed as *lov* gene for clarification purposes. We performed an *in silico* analysis of the *lov* gene using the reported sequence of *Xanthomonas axonopodis* pv. citri str. 306 (accession number AE008923.1). We identified the putative −35 and −10 promoter sequences of this gene and found a XVM2 element located 329 bp upstream of the start codon. This element has been shown to be involved in the induction of several genes related to the pathogenesis of *X. axonopodis* pv. citri [35].

**Figure 1:**
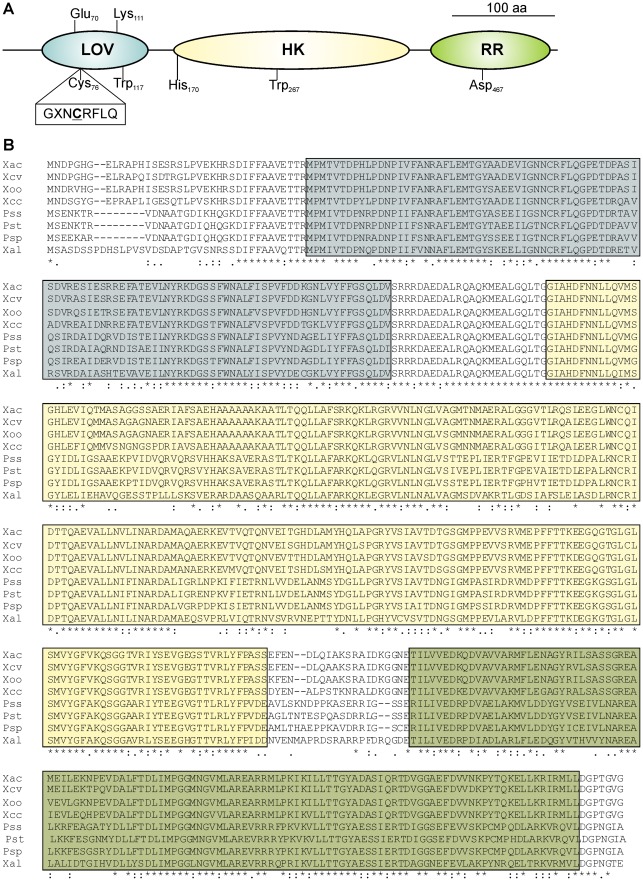
*In silico* analysis of the Xac-LOV protein. (A) Representation of the Xac-LOV protein showing the LOV domain (LOV, aa 39-142), the Histidine Kinase domain (HK, aa 167-396) and the Response-Regulator domain (RR, aa 417-533). Cys 76: amino acid involved in photoadduct formation. (B) Multiple alignments of the deduced amino acid sequences of LOV proteins from *X. axonopodis* pv. citri (Xac), *Xanthomonas campestris* pv. vesicatoria (Xcv), *Xanthomonas oryzae* pv. oryzae (Xoo), *Xanthomonas campestris* pv. campestris (Xcc), *Pseudomonas syringae* pv. syringae (Pss), *Pseudomonas syringae* pv. tomato (Pst), *Pseudomonas syringae* pv. phaseolicola (Psp) and *Xanthomonas albilineans* (Xal); performed using ClustalX [83]. The LOV, HK and RR domains are highlighted in blue, yellow and green, respectively. An asterisk indicates complete residue conservation, a colon indicates strong group conservation, a period indicates weak group conservation, and a blank space indicates no conservation of residues.

Analysis of the predicted amino acid sequence of Xac-LOV protein (540 aa) revealed the presence of a LOV domain (aa 39-142), a Histidine Kinase domain (HK, aa 167-396) and a Response-Regulator domain (RR, aa 417-533), corroborating the hybrid nature of this photoreceptor (ExPasy-Prosite proteomic server, [36]). Figure 1A shows a scale diagram of the three functional domains of the protein, with the most relevant amino acids indicated. The LOV domain contains a highly conserved motif G*X*NCRFLQ (variant Y*X*DCRFLQG) [37], [38], which includes the Cys residue responsible for the formation of a covalent adduct with the FMN chromophore in all LOV proteins studied to date [6]. When we analyzed the predicted amino acid sequence of Xac-LOV using the TM Pred software [39], no potential transmembrane domains were found. This result suggests a cytoplasmic localization for the Xac-LOV protein.

Multiple alignments of the deduced amino acid sequences of LOV proteins from several related plant pathogens including *X. axonopodis* pv. citri, *Xanthomonas campestris* pv. vesicatoria (Xcv), *Xanthomonas oryzae* pv. oryzae (Xoo), *Xanthomonas campestris* pv. campestris (Xcc), *Pseudomonas syringae* pv. syringae (Pss), *Pseudomonas syringae* pv. tomato (Pst), *Pseudomonas syringae* pv. phaseolicola (Psp) and *Xanthomonas albilineans* (Xal), revealed that the LOV proteins from these bacteria possess highly conserved LOV, HK and RR domains (Figure 1B).

### The Xac-LOV Protein Presents Canonical LOV Photochemistry

**Figure 2:**
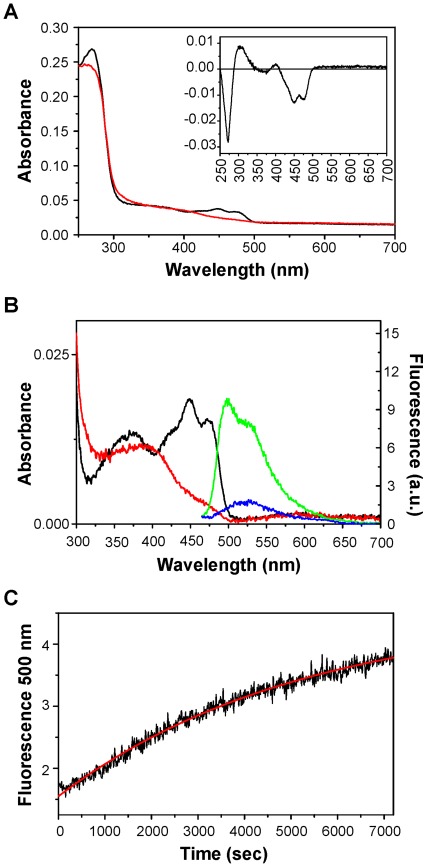
Spectroscopic analysis of recombinant Xac-LOV protein from *X. axonopodis* pv. citri. (A) Absorption spectra of dark- and light-adapted states of Xac-LOV protein (black and red lines, respectively). The inset displays the light-minus-dark difference spectrum showing the typical features of a LOV photoreceptor protein. (B) Absorption spectra of dark- and light-adapted states of Xac-LOV protein corrected for the scattering contribution (black and red lines, respectively). Fluorescence spectra (450 nm excitation) of the dark and light state of the protein are shown in green and blue lines, respectively. (C) Dark recovery kinetics of Xac-LOV recorded by monitoring the recovery of FMN fluorescence at 500 nm after excitation at 305 nm at 20°C. Fitting of the trace with a monoexponential function (red line) gave a lifetime τ_rec_ = 5200 sec. a.u.: arbitrary units.

To obtain the Xac-LOV protein for spectroscopic analysis, the *X. axonopodis* pv. citri *lov* gene was cloned in a pET-28a (+) vector and expressed in *E. coli* BL21 (DE3) Codon Plus-RIL (Stratagene). The procedure is detailed in the Supporting Information S1 and shown in Figure S1. It has been shown that following the absorption of blue light, LOV proteins generate an adduct by the formation of a covalent bond between a Cys residue from the LOV domain and the C4 carbon atom of the flavin molecule that acts as cofactor, with a two-electron reduction [12]. The adduct shows a distinct absorption spectrum, blue-shifted with respect to the unphotoactivated state, and loses its fluorescence [16]. We recorded the absorption spectra of the purified protein under dark and light conditions and obtained a light-minus-dark difference spectrum. The absorption spectrum in darkness presented the typical features of oxidized flavin chromophores with an absorption maximum at 450 nm. Following blue light illumination, we observed the loss of the absorption peak at 450 nm and the appearance of a second peak with a broad absorption band in the UVA region (Figure 2A). The light-dark difference spectrum (inset of Figure 2A) shows a canonical LOV photochemistry for Xac-LOV. Figure 2B shows the absorption spectra of the dark- and light-adapted states of the Xac-LOV protein corrected for the scattering contribution. The light spectrum showed a maximum at ca 400 nm while maxima at 375, 450 and 475 nm were observed in the dark condition. We also recorded fluorescence spectra (450 nm excitation) of the darkness and light state of the protein. In dark conditions, we could observe a maximum at approximately 500 nm, characteristic of the presence of FMN in the protein, which disappeared upon blue light illumination (Figure 2B). Incomplete photoconversion (residual band around 450 nm and residual fluorescence) is due to a small fraction of free chromophore, as evidenced by the blue line showing an absorbance maximum around 530 nm, typical of free FMN. Finally, as the FMN molecule loses it fluorescence when it is part of the photoadduct and recovers it when the protein returns to its ground state, the maximum emission peak of FMN (500 nm) was used to measure the recovery time of the basal state of the protein in dark conditions after being excited with blue light (Figure 2C). The recovery of the basal kinetic state fitted to a mono exponential function gave a lifetime (τ_rec_) of 5200 sec at 20°C.

### Physiological Characterization of a *X. axonopodis* pv. citri *lov* Mutant

To evaluate the role of the Xac-LOV protein in bacterial physiology and during the interaction between *X. axonopodis* pv. citri and host plants, we constructed a *lov* deletion mutant named Δ*lov*. We also constructed the complemented strain, named Δ*lov-*p*lov*. Details of the construction are explained in the Supporting Information S1 and shown in Figure S2. The viability and growth rate of these strains were comparable to those of the *X. axonopodis* pv. citri wild-type (WT) strain, as shown in Figure S3. We also tested the expression of Xac-LOV protein by western blot analysis using polyclonal anti-Xac-LOV rabbit antibodies (detailed on Supporting Information S1 and Figure S2Cii). We observed an inmunoreactive band for *X. axonopodis* pv. citri WT and complemented strains, but this band was absent in the mutant strain. A more intense band was obtained for the complemented strain compared to the WT despite the same amount of protein extracts being used for the western blot assay. This result is probably due to the low but still multiple copy number of the plasmid used for the complementation.

### The Deletion of the *lov* Gene Alters *X. axonopodis* pv. citri Motility

**Figure 3:**
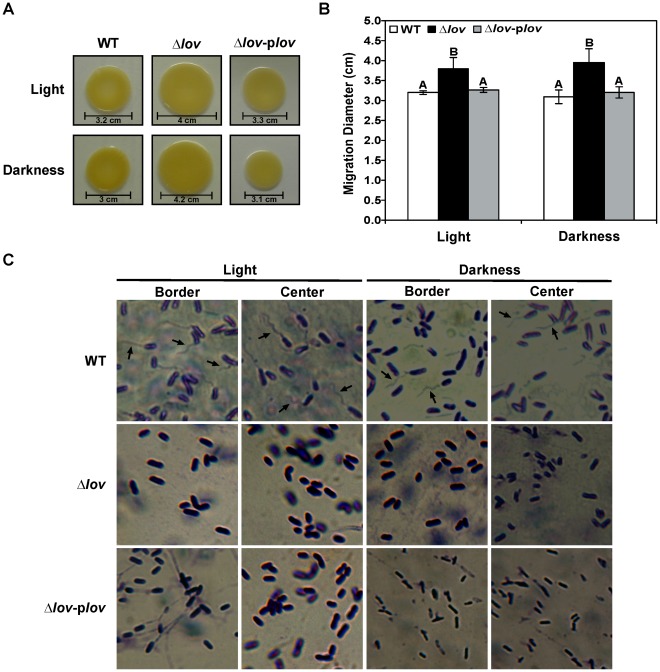
Swarming motility of *X. axonopodis* pv. citri strains. *X. axonopodis* pv. citri WT, Δ*lov* and Δ*lov*-p*lov* strains were grown on SB-0.7% w/v agar plates at 28°C for three days under light and dark conditions. (A) Direct observation of the migration zones. (B) Measurement of migration zones diameters. Data are represented as the mean +/− standard error of three independent biological samples and different letters above the bars indicate significant differences between the corresponding data (p<0.01). (C) Bacteria from the center and border regions of the migration zones were examined under a light microscope at a 1000X magnification. Arrows indicate bacterium flagella.

Bacterial motility is a very important attribute for pathogenic and non-pathogenic bacteria, which allows the colonization of nutrient-rich surfaces and host tissues. Swarming motility is a coordinated translocation of a bacterial population across solid or semi-solid surfaces and depends on flagella and the secretion of exopolysaccharide [34]. We studied swarming motility by inoculation of *X. axonopodis* pv. citri WT, Δ*lov* and Δ*lov-*p*lov* strains in SB-0.7% w/v agar plates. After three days of bacterial growth at 28°C, the Δ*lov* strain migrated further than the WT and complemented strains, both in light and dark conditions (Figure 3A). The diameters of the migration zones confirmed the plate phenotype (Figure 3B), showing statistically significant differences between Δ*lov* and WT or complemented strains, but not between growing conditions (light/darkness).

To determine potential differences on the synthesis of flagellin, the major flagellum component, we collected samples from the migration zones generated by *X. axonopodis* pv. citri WT, Δ*lov* and Δ*lov-*p*lov* under the two light conditions for protein extraction. Protein extracts were adjusted to the same protein amount and loaded onto a polyacrylamide gel to perform a western blot analysis using polyclonal anti-flagellin antibodies from *Serratia marcesens*. We observed an immunoreactive band corresponding to flagellin for *X. axonopodis* pv. citri WT in both growth conditions, but that band was hardly detected for the mutant and complemented strains (detailed on Supporting Information S1 and Figure S4). In addition, we stained the samples obtained from the border and center regions of the migration zones for flagella visualization. Figure 3C shows the microscopic images of the three *X. axonopodis* pv. citri strains. Pictures were taken at random regions under the microscope. The presence of flagella was observed for *X. axonopodis* pv. citri WT in light and dark conditions, while no flagella were observed for the mutant and complemented strains. We performed a minimum of three independent experiments, obtaining the same results.

**Figure 4:**
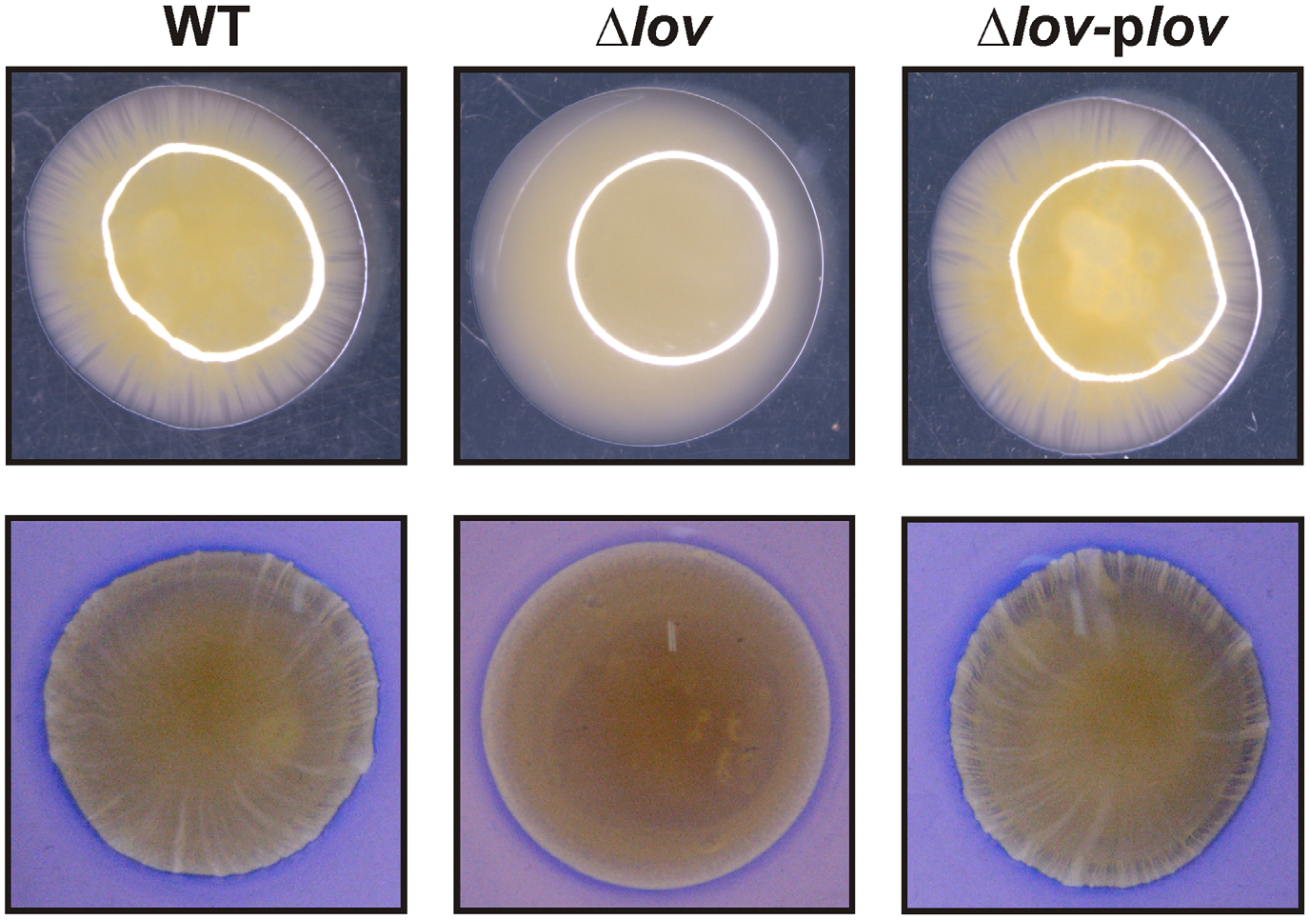
Twitching-like motility of *X. axonopodis* pv. citri strains. *X. axonopodis* pv. citri WT, Δ*lov* and Δ*lov*-p*lov* strains were stab-inoculated on SB-1% w/v agar plates and grown for two days at 28°C. To analyze the borders of the migration zones, the plates were observed under a magnifying glass (10X), prior (upper panels) and after (lower panels) staining with Coomassie Brilliant Blue R250.

Twitching motility is a type of bacterial translocation over moist surfaces mediated by the extension, attachment and retraction of type IV pili, previously described for several pathogenic bacteria [40], [41]. In order to evaluate the possibility of such migration mechanism we assayed the *X. axonopodis* pv. citri behavior in conditions known to favor twitching motility [40]. When we analyzed the bacterial migration zones obtained two days after stab-inoculating *X. axonopodis* pv. citri WT, Δ*lov* and Δ*lov*-p*lov* strains onto SB-1% w/v agar plates, we observed that WT and Δ*lov*-p*lov* colonies showed an irregular appearance with long bacterial extensions irradiating from the migration zones, which resemble the bacterial rafts showed by some twitching-performing bacteria (Figure 4) [41]. Conversely, the Δ*lov* strain developed smooth-margined colonies, with no visible bacterial extensions radiating from the central bacterial colony. Similar results were obtained when bacteria were grown in the darkness (detailed on Supporting Information S1 and Figure S5).

### The Absence of the *lov* Gene Alters Colony Morphology and Increases Exopolysaccharide Production

**Figure 5:**
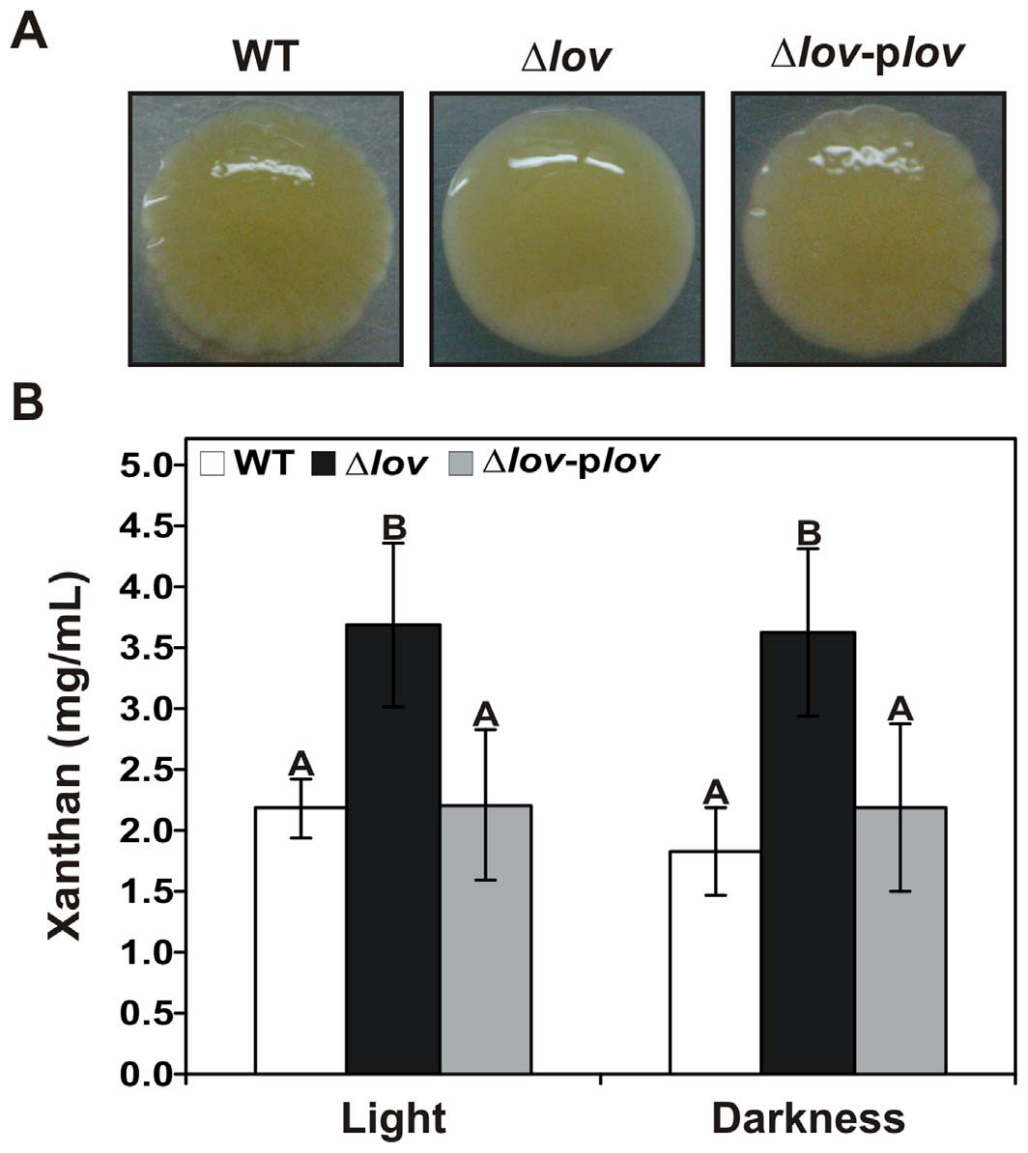
Colony morphology and exopolysaccharide production of *X. axonopodis* pv. citri strains. (A) *X. axonopodis* pv. citri WT, Δ*lov* and Δ*lov*-p*lov* colonies were analyzed on SB-1.5% w/v agar plates supplemented with 4 g/L glucose. (B) Xanthan was precipitated and quantified from bacterial culture supernatants after two days of bacterial growth in light and dark conditions. Data are represented as the mean +/− standard error of three independent biological samples and different letters above the bars indicate significant differences between the corresponding data (p<0.01).

*Xanthomonas* spp. produce a characteristic extracellular polysaccharide (EPS) named xanthan, which is responsible for the mucoid (mucus-like) appearance of bacterial colonies developed in a solid medium [42], [43]. To study possible modifications on the cellular morphology of the *X. axonopodis* pv. citri Δ*lov* strain, we analyzed the bacterial colonies developed on SB-1.5% w/v agar plates supplemented with 4 g/L glucose. We observed that Δ*lov* colonies had a more glossy and mucoid appearance than WT and Δ*lov*-p*lov* colonies. Moreover, while the colonies of the WT and complemented strains of *X. axonopodis* pv. citri showed an irregular surface with scalloped margins, the colonies of the Δ*lov* strain were straight and smooth (Figure 5A). Bacteria were also grown on SB-agar plates supplemented with Congo red, a dye that strongly interacts with external polysaccharides. This dye is widely used for the detection of curly fibers, thin aggregative fibers present in several microorganisms [44], [45]. Although the morphology differences described above were maintained, there were no differences in Congo red binding between *X. axonopodis* pv. citri strains (detailed on Supporting Information S1 and Figure S6). On the basis of these observations we quantified xanthan from the supernatants of two-day-old cultures of *X. axonopodis* pv. citri WT, Δ*lov* and Δ*lov*-p*lov*, and found that both in light and dark conditions, the Δ*lov* strain produced a statistically significant higher amount of EPS compared to the WT or the complemented strains (Figure 5B).

We also assessed bacterial survival in the presence of hydrogen peroxide and found that the Δ*lov* strain was three-fold more sensitive to these treatments compared to the WT strain, suggesting a potential protective role of *lov* gene upon oxidative stress. The level of survival of the Δ*lov*-p*lov* strain resulted intermediate between the WT and Δ*lov* strains (detailed on Supporting Information S1 and Figure S7).

### Biofilm Formation is Affected by the Deletion of the *lov* Gene

**Figure 6:**
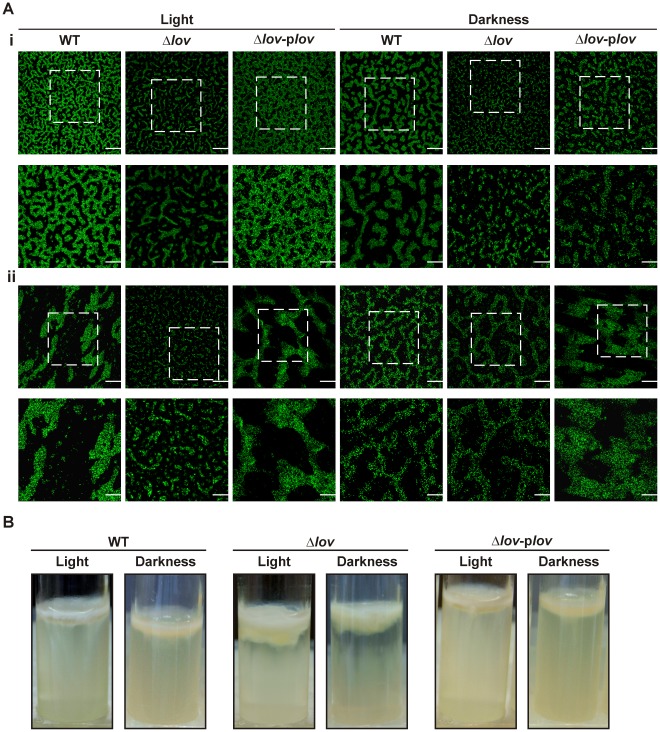
Biofilm formation by *X. axonopodis* pv. citri strains. (A) Green fluorescent protein (GFP)-labeled bacteria were grown on chambered cover slides and visualized under confocal laser scanning microscopy (CLSM) after two (i) and five (ii) days of bacterial growth. For each time period, the upper panels show the biofilms developed at the bottom of the chambered cover slides with a magnification of 400X, and the bottom panels show a 2X zoom of the regions marked in the previous panels. Scale bars, 50 µm. In this experiment Δ*lov*-p*lov* corresponds to the Δ*lov* strain complemented with pBBR-p*lov*2 (Δ*lov*-p*lov*’). (B) *X. axonopodis* pv. citri strains were statically grown on glass tubes for two weeks at 28°C. Biofilms were observed on the air-liquid interface. In each case, bacteria were grown under light and dark conditions.

Biofilm formation is associated with the production of exopolysaccharide and is important for the virulence of some pathogenic bacteria because it can promote their survival against the action of antimicrobial compounds derived from host organisms [46]. We used confocal laser scanning microscopy (CLSM) to analyze the morphology of bacterial biofilms developed by green fluorescent protein (GFP)-labeled strains of *X. axonopodis* pv. citri on chambered cover glass slides over different periods of time. The *X. axonopodis* pv. citri WT, Δ*lov* and complemented strains were able to develop complex structures consisting of clustered bacteria in close contact with each other. However, as shown in Figure 6A, we observed different patterns of bacterial aggregation between the different strains. After two days of incubation under light conditions, the three strains generated isolated microcolonies in which bacteria were densely packed and organized, maintaining lateral interactions with each other, however these structures were larger for the WT and complemented strains. After five days, the WT and complemented strains generated aggregates that extended over the entire surface, while the microcolonies developed by the Δ*lov* strain remained disperse. In dark conditions, after two days of incubation, the pattern of cell aggregation of the WT and complemented strains was similar to that observed for Δ*lov* under light conditions. However, the Δ*lov* strain generated even smaller structures that were considerably less organized than those generated under light conditions. After five days, cell aggregation patterns were rather similar for the three strains with the formation of a cellular matrix that filled the entire surface of the chamber bottom.

We also assayed the ability of *X. axonopodis* pv. citri to develop a biofilm on glass tubes containing SB liquid medium, in order to evaluate its aggregating ability after longer incubation times. After two weeks of static incubation at 28°C in different illuminating conditions, we observed cell and EPS aggregates on the air-liquid interface. In the case of the Δ*lov* strain, we observed highly flocculated aggregates that maintained a limpid solution sinus. In contrast, the WT and complemented strains showed less dense aggregates on the interface with a turbid solution sinus (Figure 6B). For both assays we performed a minimum of three independent experiments with the same results.

### The Absence of the *lov* Gene Affects *in vitro* Adhesion of *X. axonopodis* pv. citri

**Figure 7:**
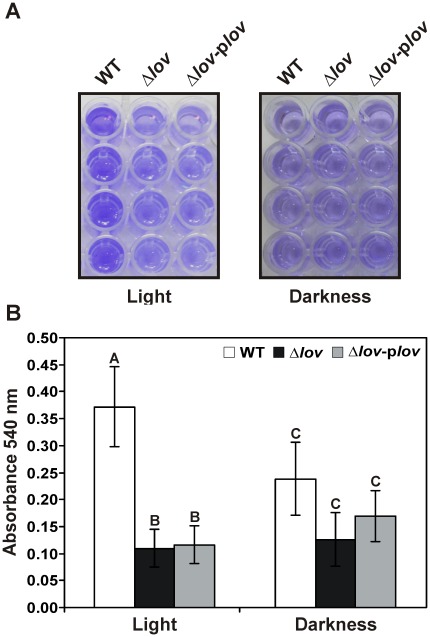
Adhesion of *X. axonopodis* pv. citri strains to an abiotic surface. (A) *X. axonopodis* pv. citri WT, Δ*lov* and Δ*lov*-p*lov* strains were grown in XVM2 medium, centrifuged, resuspended in the same medium, and 100 µL of the bacterial suspensions were placed in the wells of a 96-well polyvinyl chloride (PVC) plate. After incubation at 28°C for 6 h under light and dark conditions, surface-attached cells were stained with 1% w/v Crystal violet. (B) The attached dye was solubilized with 95% ethanol, transferred to an eppendorf tube and quantified by absorbance at 540 nm. Data are represented as the mean +/− standard error of three independent biological samples and different letters above the bars indicate significant differences between the corresponding data (p<0.01).

The ability to adhere to host tissues is essential for successful infection by many microbial pathogens [43]. To analyze this phenomenon, we studied the ability of *X. axonopodis* pv. citri strains to adhere to abiotic and biotic surfaces. Assays were performed by growing bacteria in XVM2 medium, a minimum medium that simulates conditions of the apoplastic space [47], [48]. For the abiotic assay, we employed polyvinylchloride (PVC) plates on which bacteria were grown for six hours at 28°C. After washing and staining the plates with Crystal violet, we observed that under light conditions, the adhesion of the Δ*lov* strain was diminished compared to the WT strain (Figure 7A). The adhesion of the complemented strain was similar to that of the mutant strain. On the other hand, when the assay was performed under dark conditions, all of the *X. axonopodis* pv. citri strains presented a reduced adhesion compared to adhesion in the light condition. Bacterial attachment measured by the spectroscopic quantification of the bound dye at 540 nm confirmed that in the presence of light, the adhesion of Δ*lov* and Δ*lov*-p*lov* was statistically significant lower than that of the WT (Figure 7B). In contrast, under dark conditions, there were no significant differences between the adhesion of the different *X. axonopodis* pv. citri strains.

### Xac-LOV is Crucial for Bacterial Adhesion to Orange Leaves

**Figure 8:**
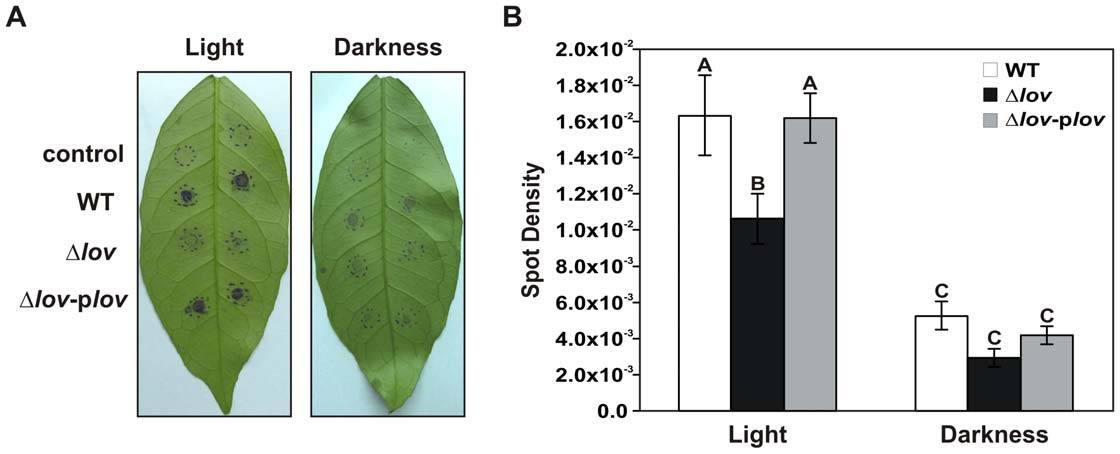
Adhesion of *X. axonopodis* pv. citri strains to orange leaves. (A) *X. axonopodis* pv. citri WT, Δ*lov* and Δ*lov*-p*lov* strains were grown in XVM2 medium, cultures were centrifuged, resuspended and placed on the abaxial face of orange leaves. After incubation at 28°C for 6 h under light and dark conditions, surface-attached cells were stained with 0.1% w/v Crystal violet dye. Control: XVM2 medium. The order of inoculation is indicated at the left of the panel. Dashed lines in the leaves indicate the inoculated area (B) Adhesion was quantified by image analysis determining the spot density (intensity/pixel area) of each adhesion region. Data are represented as the mean +/− standard error of three independent biological samples and different letters above the bars indicate significant differences between the corresponding data (p<0.01).

To study the adhesion of *X. axonopodis* pv. citri strains to biotic surfaces bacteria were grown in XVM2 medium and placed on the surface of orange leaves. After six hours of incubation at 28°C, the leaves were washed and stained with Crystal violet. When the assay was performed under light conditions, the adhesion ability of the *X. axonopodis* pv. citri Δ*lov* strain was much lower than for the WT and complemented strains (Figure 8A). In the dark condition, the adhesion of all *X. axonopodis* pv. citri strains was very low. Bacterial attachment was quantified by digital image analysis (spot density) of the stained leaves (Figure 8B). This quantification demonstrated that under light conditions the adhesion of the Δ*lov* strain was statistically significant lower than that of the WT or complemented strains. In darkness, the differences in the adhesion of the three strains were not statistically significant, but we found a significant reduction in the adhesion of the three *X. axonopodis* pv. citri strains compared to the corresponding adhesion in the light condition.

Bacterial adhesion is highly related with the presence of adhesins. When we evaluated the expression of the *fhaB* gene, encoding a filamentous hemagglutin-like adhesin, we could observe that the Δ*lov* strain presented a reduced expression of this gene compared to the WT and Δ*lov*-p*lov* strain (detailed on Supporting Information S1 and Figure S8).

### The Deletion of the *X. axonopodis* pv. citri *lov* Gene Modifies Plant Disease Symptoms

**Figure 9:**
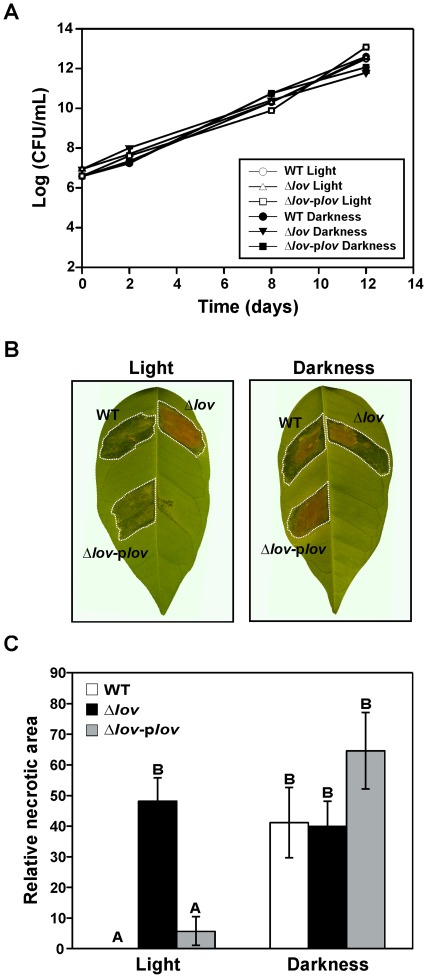
*In planta* growth and disease symptoms. (A) *X. axonopodis* pv. citri WT, Δ*lov* and Δ*lov*-p*lov* cultures were adjusted to 10^7^ colony forming units (CFU)/mL in 10 mM MgCl_2_ and used to infiltrate orange leaves. The inoculated leaves were maintained either in a 16 h light/8 h dark photoperiod (light), or covered with cardboard envelopes (darkness). Leaf samples were taken at different times after inoculation, 0.8 cm diameter discs were ground in 100 µL of 10 mM MgCl_2_ and after serial dilution samples were plated for colony counting. Results were expressed as Log CFU/mL. (B) Symptoms developed in the infiltrated leaves two weeks after inoculation with the bacterial cultures. (C) Tissue damage was quantified by digital image analysis of the infiltrated leaves. Results were expressed as relative necrotic area 100 X [necrotic area (brown) pixels/total infiltrated area (dotted line) pixels]. Data are represented as the mean +/− standard error of three independent biological samples and different letters above the bars indicate significant differences between the corresponding data (p<0.01).

The ability of *X. axonopodis* pv. citri strains to develop disease symptoms in *Citrus sinensis* (orange) leaves was studied by inoculation of the leaves with cultures of WT, Δ*lov* and Δ*lov*-p*lov* strains adjusted to 10^7^ colony forming units (CFU)/mL. The infiltrated leaves were maintained in conventional photoperiod (light condition) and in the darkness. We analyzed the bacterial growth at different times after inoculation of the leaves, and observed that it was similar for the three *X. axonopodis* pv. citri strains until two weeks after inoculation (Figure 9A). In contrast, the symptoms caused by the mutant strain were phenotypically different than those generated by the WT and complemented strains. In the light condition, while the tissue regions inoculated with the WT and complemented strains showed typical canker lesions, the region inoculated with the Δ*lov* strain presented a highly necrotic appearance. In the dark condition the aspect of the lesions generated by the three *X. axonopodis* pv. citri strains resulted similar, with a high level of necrosis in the inoculated tissue (Figure 9B). We quantified the degree of necrosis developed in the orange leaves inoculated with the different bacterial strains as the rate between the necrotic area and the total inoculated area, corroborating the direct observation (Figure 9C).

## Discussion

Prokaryotes have evolved a repertoire of photosensory proteins that detect the visible light environment to regulate cell physiology. Diverse classes of prokaryotic photoreceptors with different regulatory roles have been recently identified and several reports on heterotrophic bacteria have revealed physiological functions regulated by blue light receptors [5]. The *X. axonopodis* pv. citri genome has three putative blue light photoreceptors, two with BLUF domains and one with a LOV domain [31], [32]. The high representation of these genes in *X. axonopodis* pv. citri led us to suggest a probable relevance of blue light for its survival and for the colonization of host plants. The Xac-LOV protein consists of three distinct domains: a LOV domain, a HK domain and an RR domain. This protein has several conserved key functional amino acid residues known to be important for photochemistry and signaling (Figure 1). Many of the studied LOV domains are found together with HK motifs. The organization of hybrid LOV-HK-RR proteins is found almost exclusively in bacterial plant pathogenic species such as Xcv, Xoo, Xcc, Pss, Pst, Psp and Xal [14], [25]. Other bacterial LOV proteins with associated HK activity are those from *B. melitensis, B. abortus* and *Erythrobacter litoralis*, but these proteins do not include the RR domain [7]. All of these sensor/HK proteins are hypothesized to be involved in blue light-driven two-component signaling systems at some stage of the bacterial life cycle [49], [50]. The LOV proteins present in some pathogenic bacteria, such as *P. syringae* and *B. melitensis,* were reported to be induced in conditions known to promote bacterial virulence [7], [26]. Accordingly, we found that the promoter region of the *lov* gene includes a regulatory XVM2 element known to be involved in the induction of several genes related to the pathogenesis of *X. axonopodis* pv. citri [35], [48]. All of these results suggest that LOV proteins are likely involved in bacterial virulence.

We evaluated the ability of the Xac-LOV protein to sense and respond to blue light. Studies of absorption and fluorescence spectroscopy using a recombinant Xac-LOV protein showed that facing blue light illumination, this protein has spectral variations typical of the formation of a photoadduct (Figure 2A, B). The spectral properties of the Xac-LOV protein are consistent with those exposed by several bacterial LOV proteins and by plant phototropins [16], [17], [51], demonstrating that Xac-LOV is in fact activated by blue light. Most LOV domains complete a photocycle from the photoadduct back to the ground state in the darkness, with half-lives of seconds to several minutes [52], [53]. In the case of Xac-LOV, the photoadduct showed a very slow rate of reversion to the ground state in the darkness (τ_rec_ 5200 s) (Figure 2C). These results are consistent with those presented by Cao *et al*., who showed that the Pst LOV protein has the typical LOV photochemistry with a recovery time in the darkness of 5650 s [27]. Differences in the kinetics of adduct formation and rupture among LOV proteins can be explained by variations in the residues that make up the binding pocket of the flavin cofactor [12], [54], [55] and in more distant amino acids [14], [56].

To evaluate the potential role of Xac-LOV protein in bacterial physiology and host plant colonization, we constructed a *lov* deletion mutant (Δ*lov* strain). The analyses of growth curves in liquid medium indicated that the *lov* gene is not essential for *X. axonopodis* pv. citri viability (Figure S3).

Bacteria use a variety of motility mechanisms to colonize host tissues. These mechanisms include flagella-dependent swimming and swarming and flagella-independent twitching, gliding and sliding [34]. Previous work from our laboratory demonstrated that *X. axonopodis* pv. citri exhibits swarming motility and that this motility depends on flagella and EPS secretion into the medium [57]. In the present study, we observed an increased motility for the Δ*lov* strain compared to the WT and complemented strains, both in light and dark conditions (Figure 3A, B). These results suggest that the *lov* gene may be a component of the bacterial motility regulation network. *X. axonopodis* pv. citri possess a single polar flagellum, which is composed of multiple units of flagellin protein, encoded by the *fliC* gene [31], [58]. Western blot analysis revealed a decreased flagellin content in the Δ*lov* strain in both lighting conditions tested (Figure S4). Accordingly, by optical microscopy analysis, we observed a lower number of flagellar structures in the mutant strain of *X. axonopodis* pv. citri compared to the WT strain (Figure 3C). These results suggest a role of the *lov* gene in flagella development. Although the complemented strain showed a reversion of the lower migration phenotype, we could not observe the recovery of the WT levels of flagella in this strain. The fact that no complementation was observed for flagella synthesis could be a consequence of the lack of fine control in the expression of the *lov* gene on the complemented strain. Although this strain was constructed by introducing the *lov* gene under the control of its own promoter on a low copy vector, the expression levels of the *lov* gene are not necessarily identical to the normal expression levels of this gene in the WT strain. In fact, a western blot analysis showed a higher *lov* expression for the Δ*lov*-p*lov* strain compared to the WT strain (Figure S2Cii). As reported for other bacterial species LOV proteins act at the first level of the signal transduction process affecting several bacterial features [5]. It is possible that some of these features (such as flagella synthesis) require a tightly controlled level of the regulator proteins while for other processes a wider range of the regulator protein level is adequate to maintain the normal behavior. While the level of expression of the *lov* gene provided by the vector used for complementation was adequate to achieve the complementation of some physiological aspects, such as EPS production, it is possible that regulation of flagella synthesis requires a more tightly regulated level of the Xac-LOV protein.

We also studied bacterial motility in conditions that favor twitching, a flagella-independent mechanism of bacterial translocation over moist surfaces directed by polar type IV pili and characterized by the formation of bacterial rafts on the colony borders [41]. We found that the *X. axonopodis* pv. citri strains behave differently on twitching plates. While WT and Δ*lov*-p*lov* colonies exhibited irregular borders, Δ*lov* colonies showed smooth edges in the migration zones (Figure 4). These results suggest that *X. axonopodis* pv. citri can perform a twitching-like motility, and that the *lov* gene has a role in this type of translocation.

Another bacterial feature important for host plant colonization is the production of EPS. *Xanthomonas* spp. produce a major EPS named xanthan, which was previously reported to be important for epiphytic survival [42], swarming motility [57] and biofilm formation [59] of *X. axonopodis* pv. citri. We found that the Δ*lov* strain produces more EPS than the WT and complemented strains (Figure 5). This result is consistent with the increased bacterial motility of the Δ*lov* strain despite the reduction of flagella [46], [57]. When we evaluated the interaction of Congo red stain, which interacts with extracellular components such as curly fibers [44], no differences were found between the different *X. axonopodis* pv. citri strains, indicating no modifications in these structures (Figure S6).

Biofilms are microbial communities immersed in a self-produced exopolysaccharide matrix and attached to an inert or living surface [60]–[62]. Several reports indicate that biofilms are important for the virulence of many pathogenic bacteria [46], [63]. In *X. axonopodis* pv. citri, the EPS and flagellum have been associated with the formation of mature biofilms [59], [64]. When we studied early biofilm formation by CLSM, we could observed that the *X. axonopodis* pv. citri WT, mutant and complemented strains were able to develop complex cellular structures, however these structures were smaller and more dispersed for the Δ*lov* strain (Figure 6A). When we analyzed the bacterial aggregation on the air-liquid interface of glass tubes after a static incubation of *X. axonopodis* pv. citri strains for two weeks, we observed thicker cellular aggregates for the Δ*lov* strain compared to the WT and complemented strains (Figure 6B). We hypothesized that the Δ*lov* strain could have impaired the early developmental stage of biofilm formation, but after longer incubation times its aggregating ability results higher than for the WT strain. This phenomenon can be explained by the major involvement of different structures on the different stages of biofilm formation. Therefore, considering that flagellum-dependent motility and bacterial attachment are essential for the initiation of biofilm development, the decrease in flagellum synthesis and the low adhesion ability observed for the Δ*lov* strain of *X. axonopodis* pv. citri can explain the initial impairment of biofilm formation of this strain (65,66). On the other hand, the secretion of exopolysaccharide is known to be important for late stages of biofilm formation (establishment of mature biofilms) [65], [66]. In this context, the increased levels of xanthan measured for the Δ*lov* strain are consistent with the larger bacterial aggregates observed for this strain after longer incubation periods.

Bacterial attachment is a crucial early step in the pathogenicity process. We evaluated the adhesion capacity of the different *X. axonopodis* pv. citri strains to abiotic and biotic surfaces employing XVM2 as a growth medium, known to improve the expression of adhesins genes [57]. In both assays we found that the adhesion ability of *X. axonopodis* pv. citri was reduced by the deletion of the *lov* gene, and that the adhesion of the WT strain was diminished in the darkness (Figures 7 and 8). These results suggest that *X. axonopodis* pv. citri adhesion requires a functional *lov* gene and that the attachment process is dependent on the presence of light. Our results are similar to those obtained in *Caulobacter crescentus*, where it was demonstrated that light regulates cell-surface attachment through a LOV protein [23], [67]. The fact that the adhesion of the Δ*lov* strain was also reduced in darkness compared to light suggests an additive effect on bacterial adhesion. This could be explained by the simultaneous participation of Xac-LOV and other *X. axonopodis* pv. citri photosensory proteins in the regulation of bacterial attachment. It should be noted that in addition to the *lov* gene encoding the LOV protein, the *X. axonopodis* pv. citri genome contains other putative photoreceptors, i.e., two genes encoding BLUF proteins and one encoding a phytochrome [31], [32]. Several factors can contribute to bacterial adhesion, namely fimbrial and non-fimbrial adhesins, EPS and flagella [68]–[72]. In a previous study, it was demonstrated that plant tissue attachment is dependent on the coordinated action of specific adhesins and EPS, and that *X. axonopodis* pv. citri grown in XVM2 shows minor EPS production [57]. This suggests that the differences in adhesion between *X. axonopodis* pv. citri WT and Δ*lov* could be a consequence of alterations in bacterial adhesins. The expression of the *fhaB* gene, encoding a filamentous hemagglutin-like protein known to be involved in the bacterial adhesion to abiotic surfaces and host tissues [57], resulted reduced for the Δ*lov* strain compared with the WT strain. This result can explain the decrease of the adhesion ability of the Δ*lov* strain.

When we performed the assays with the Δ*lov*-p*lov* strain, we only observe a reversion to the WT phenotype in the adhesion to orange leaves. As mentioned above, the main factors that contribute to the bacterial adhesion to abiotic and biotic surfaces are adhesins, EPS and flagella [68]–[72]. Taking in consideration that the complemented strain showed a reversion to the *fhaB* adhesin gene expression, but was not able to restore the synthesis of flagella, the absence of complementation during the *in vitro* adhesion assay could be due to the absence of this structure in the Δ*lov*-p*lov* strain. The different outcome for the adhesion of the complemented strain during the *in vitro* and *in planta* assay could be a consequence of the major involvement of different factors during the adhesion to the diverse surfaces, possibly being the bacterial flagellum more relevant for the adhesion to abiotic surfaces, and the adhesins mainly involved in the adhesion to host tissues.

*X. axonopodis* pv. citri is an obligate aerobic phytopathogen and consequently, is exposed to hydrogen peroxide produced by normal aerobic respiration and as a key component of the host immune response [73]. Pathogens need to prevent and overcome oxidative stress to establish and maintain infections [74]. We found that the Δ*lov* strain presents an increased sensitivity to oxidative stress (Figure S7), suggesting a potential participation of the product of the *lov* gene in the protective mechanisms triggered upon this type of stress. Since intense blue light can result in oxidative damage to cells by the light-driven formation of reactive oxygen intermediates [75], it could be advantageous for the bacterium to sense this condition to activate appropriate defense systems.

Finally, we analyzed the role of the Xac-LOV protein in the interaction between *X. axonopodis* pv. citri and host plants, and we observed that the virulence process was affected in the Δ*lov* mutant. The orange leaves infected with the mutant strain displayed phenotypically different lesions than those rendered by the WT and complemented strains, generating visible necrotic regions on the infected tissues. This result suggests the participation of the Xac-LOV protein in the regulation of the virulence process, likely preventing excessive tissue necrosis. The modulation of tissue damaging is a mechanism that allows *X. axonopodis* pv. citri, a hemibiotrophic pathogen, to remain in the infected tissue long enough to proliferate and to spread to neighboring tissues [76]. In this context, the *lov* gene could be involved in the control of the host tissue damage caused by the phytopathogen. The loss of the control of tissue damage during the infection performed in darkness reflects the light-dependent nature of this process. Studies performed with *B. abortus* and *B. mellitensis* revealed a similar role for LOV photoreceptors, as the LOV proteins present in these pathogens were found to be implicated in the pathogenesis process [7]. It is worth mentioning that the complemented strain of *X. axonopodis* pv citri was able to restore the normal appearance of the light lession, in spite of its inability to restore the normal flagella levels. This result is consistent with the one obtained by Malamud *et al*., in which a mutant strain of *X. axonopodis* pv citri unable to synthetize flagellin, exhibited an only slightly reduced pathogenicity compared to the wild-type *X. axonopodis* pv. citri strain [64].

We have alternative hypothesis to explain the fact that the complemented strain of *X. axonopodis* pv. citri was able to restore some, but not all of the processes studied in this work. First, as we mentioned above, the higher expression of the *lov* gene in the complemented strain compared to the WT strain could be unfavorable for the reversion of features such as flagellum synthesis, which require a fine control of the signaling molecules. On the other hand, keeping in mind that *X. axonopodis* pv citri presents other photoreceptor proteins including two blue light-sensing BLUF proteins and one red light-sensing phytochrome, we cannot rule out the involvement of these proteins in the regulation of some of the light-dependent mechanisms discussed in this work. Further studies with bacterial mutants in those photoreceptors will help us uncover the entire light regulated mechanism of the lifecycle and virulence of this pathogen.

In conclusion, we demonstrated that the *X. axonopodis* pv. citri *lov* gene encodes a functional photoreceptor protein that is activated by blue light, generating a signaling state that is probably involved in a downstream activation cascade. Moreover, we found that bacterial motility, EPS production, biofilm formation and adhesion are among the processes influenced by the product of the *lov* gene. Because the Xac-LOV protein is likely located at the first level of a signal transduction pathway, it is expected that this protein controls downstream components involved in more than one physiological feature. Although many of the studied physiological aspects showed a clear dependence on the *lov* gene, several of them did not have an apparent light regulation. This phenomenon could imply that other stimuli may be involved in the modulation of Xac-LOV activity. Regarding this, it has been shown that the photoactivity of some LOV and BLUF proteins can be regulated by the redox balance of the bacterial cytoplasm [77] or by temperature [18]. However, a clear light-dependent regulation was shown for *X. axonopodis* pv. citri adhesion to orange leaves, suggesting that the environmental light could modulate this process through the Xac-LOV protein. As bacterial attachment is crucial for the successful colonization of host plant tissues, this result suggests that the Xac-LOV protein and the light environment play an important role during *X. axonopodis* pv. citri host colonization. More significantly, the evaluation of the disease symptoms caused by the *lov* deletion mutant demonstrated that light and the Xac-LOV protein have an essential role in the virulence process, being involved in the control of the host tissue damage caused by the phytopathogen. It is worth mentioning that this report revealed the novel contribution of a photosensory system in the physiology of a phytopathogenic bacterium. Furthermore, this is the first report of a functional blue light receptor in *Xanthomonas* spp. and the first genetic evidence of a bacterial LOV protein involved in the control of bacterial virulence during citrus canker disease.

## Materials and Methods

### Plasmids, Bacterial Strains and Growth Conditions

The bacterial strains and plasmids used in this study are listed in Table 1. *X. axonopodis* pv. citri cells were grown aerobically at 28°C with shaking at 200 rpm in Silva Buddenhagen (SB) medium [78] or in the minimal medium XVM2 [48] supplemented with the corresponding antibiotics. All *X. axonopodis* pv. citri strains were derivatives of the strain Xcc 99–1330, which was kindly provided by Blanca I. Canteros. *E. coli* cells were grown aerobically at 37°C with shaking at 250 rpm in Luria Bertani (LB) medium [79]. Antibiotics were used at the following final concentrations: ampicillin (Amp), 25 µg/mL for *X. axonopodis* pv. citri and 100 µg/mL for *E. coli*; kanamycin (Km), 40 µg/mL for both bacteria; chloramphenicol (Cm), 20 µg/µL for *E. coli*; streptomycin (Sm), 50 µg/mL for *X. axonopodis* pv. citri and 100 µg/mL for *E. coli*; gentamycin (Gm), 40 µg/mL for *X. axonopodis* pv. citri.

**Table 1:** Bacterial strains and plasmids.

Bacterial strains/plasmids	Relevant characteristics	Reference
*Escherichia coli*		
JM109	e14^-^(MCRA^-^) *recA*1 *endA*1 *gyrA*96 *thi*1	[79]
	*hsdR*17 (*r_k_^-^m_k_^-^*) *supE*44 *relA*1 Δ(*lac-*	
	*proAB*)/F’ [*traD*36 *proA^+^ proB* ^+^	
	*lacI* ^q^ΔM15]	
BL21(DE3) Codon Plus-RIL	*argU* (AGA, AGG), *ileY* (AUA), *leuW*	Stratagene
	(CUA), (Cm^R^)	
	294::[RP4-2(Tc::Mu) (Km::Tn7)] *pro res*	
S17-1	Δ*rec*A, Tp^r^, mod^+^ Sm^r^, Sp^r^	[84]
*Xanthomonas axonopodis* pv. citri		
Xcc99-1330	Wild type, Amp^r^	Canteros
Δ*lov*	*lov* mutant of Xcc99-1330, Sm^r^	This work
Δ*lov*-p*lov*	Δ*lov* complemented with pBBR-p*lov* Sm^r^	This work
	Gm^r^	
Δ*lov*-p*lov*’	Δ*lov* complemented with pBBR-p*lov*2	This work
	Sm^r^ Km^r^	
pET-28a(+)	*ori*(ColE1), lacI, ori(f1), Kan^r^, His-*tag*	Novagen
pBLUESCRIPT II KS+ (pBS)	*ori*(ColE1), lacZ, ori(f1), Amp^r^	Stratagene
pKRP13	pUC derived plasmid, Sm^r^, Sp^r^, Amp^r^	[85]
pKMobGII	pUC derived plasmid, *mob* Kan^r^ *gus*A	[86]
	*lac*Zα	
pBBR1MCS-5	Broad host-range vector, Gm^r^	[79]
pBBR1MCS-2	Broad host-range vector, Km^r^	[79]
p*lov*	*lov* cloned in pET-28a(+)	This work
pBSI	DFS fragment cloned in pBS	This work
pBSII	UFS fragment cloned in pBSI	This work
pBSIII	Sm/Sp resistance *cassette* cloned in pBSII	This work
pK-Rec*lov*	UFS and DSF fragments containing	This work
	Sm/Sp resistance *cassette* in pKMobGII	
pBBR-p*lov*	*X. axonopodis* pv. citri *lov* gene cloned in	This work
	pBBR1MCS-5	
pBBR-p*lov*2	*X. axonopodis* pv. citri *lov* gene cloned in	This work
	pBBR1MCS-2	

Ap: ampicillin; Km: kanamycin; Cm: chloramphenicol; Gm: gentamycin; UFS: upstream flanking sequence; DSF: downstream flanking sequence.

The assays were performed under different lighting conditions. For the light conditions, bacteria were grown on a chamber with continuous blue light (λ = 462 nm, 4.115 µE/m^2^s) provided by LEDs at 28°C. The dark condition was generated by covering flasks or plates with aluminum foil.

### Spectroscopic Analysis

Absorbance spectra were recorded with a Jasco 7850 UV/Vis spectrophotometer. Steady-state fluorescence measurements were carried out with a Perkin-Elmer LS50 luminescence spectrometer. All measurements were done at 20°C using 1 cm light-path quartz cuvettes. Photoequilibrium conditions, with the accumulation of the photoactivated state (adduct), were achieved by illuminating the sample with a blue light–emitting Led-Lenser®V8 lamp (max 462 nm) (Zweibrüder Optoelectronics, Soelingen, Germany) as previously described [80].

### Bacterial Motility Assays

To analyze swarming motility, *X. axonopodis* pv. citri strain saturated cultures were subcultured into fresh SB medium at 2% inoculum and grown to late-exponential phase (15 h) in light and dark conditions. Bacteria were harvested by centrifugation and resuspended in distilled water, adjusting the cultures to 10^7^ CFU/mL. Aliquots of 3 µL were inoculated on the center of SB-0.7% w/v agar plates and incubated at 28°C in a moist chamber in light and dark conditions [34]. The migration zones were analyzed after three days of growth.

Twitching motility was analyzed as described by Semmler *et al.* [40]. Briefly, *X. axonopodis* pv. citri strains were stab-inoculated from a two-day-old SB-1.5% w/v agar plate with a sterile toothpick through a thin (approximately 3 mm) SB-1% w/v agar layer to the bottom of the Petri dish. After incubation at 28°C for two days, the zone of motility was visualized by staining with 0.05% w/v Coomassie Brilliant Blue R250 in 40% v/v methanol-10% v/v acetic acid.

### Flagella Stain

Flagella were stained as described by Kearns and Losick [81] with some modifications. Briefly, the dye was prepared by mixing 10 parts mordant (2 g tannic acid, 10 mL 5% w/v phenol, 10 mL saturated aqueous AlKO_8_S_2_·12H_2_O) with one part 12% w/v Crystal violet in ethanol. Two-day-old swarming bacteria from the border and center regions of the migration zones were picked with a sterile toothpick and resuspended in 10 µL distilled water on a microscope slide and covered with a coverslip. The slide was propped vertically, and 10 µL of dye was applied to the top edge of the coverslip to stain the sample by capillary action. Samples were observed on an Olympus BH2 microscope and recorded with a Nikon DS-FiS camera and Nikon NIS-Elements D 2.30 image capture software.

### Colony Morphology and EPS Production

For colony morphology observation, bacteria were grown on SB-1.5% w/v agar plates containing 4 g/L glucose.

To quantify the EPS production, strains were cultured in SB broth for three days under light and dark conditions. Bacteria were harvested by centrifugation, and EPS was precipitated from the culture supernatant by the addition of two volumes of ethanol. The precipitate was vacuum filtrated and weighed [78].

### Biofilm Formation Assay

*X. axonopodis* pv. citri strains were modified to express green fluorescence protein (GFP). Briefly, the coding sequence for EGFP from pEGFP-1 (Clontech, Palo Alto, CA, U.S.A.) was digested with *BamH*I and *Xba*I and ligated in-frame with the LacZ-α-peptide of the broad-host-range vector pBBR1MCS-5 [82] previously digested with the same enzymes, rendering the plasmid pBBR1MCS-5EGFP. *E. coli* S17-1 cells harboring this plasmid were conjugated to the different *X. axonopodis* pv. citri strains, and transconjugants were selected for Gm resistance. Saturated cultures of the GFP-labeled bacteria in SB medium were adjusted to the same optical density at 600 nm (OD_600_) and diluted 1∶100 in fresh medium, and 300 µL was placed onto chamber-covered glass slides (n°155411, Lab-Tek, NUNC, Naperville. IL, U.S.A.). Chambers were statically incubated in a humidified PVC-box in light and dark conditions. Biofilm formation was visualized by confocal laser scanning microscopy (Nikon Eclipse TE-2000-E2) with a motor system and DIC/Nomarski optics and a head scan D Eclipse C1si. The images obtained were analyzed with Nikon EZ-C1 3.90 software.

Biofilm formation was also studied on glass tubes. Saturated cultures of *X. axonopodis* pv. citri in SB medium were adjusted to the same OD_600_. Subsequently, 20 µL of each bacterial culture was transferred to glass tubes containing 2 mL fresh medium and statically incubated at 28°C in light and dark conditions. Bacterial aggregates were visually examined after two weeks of incubation [45].

### *In vitro* and *in planta* Adhesion Assays

*X. axonopodis* pv. citri adhesion to abiotic surfaces was assayed on polyvinyl chloride (PVC) 96-well microplates. Overnight cultures of *X. axonopodis* pv. citri strains were harvested by centrifugation and cell pellets were washed and resuspended in fresh media to the same OD_600_. Each well was filled with the bacterial suspension or 1∶10 dilutions in a final volume of 100 µL. Wells with media and water were included as negative controls. The plates were incubated for 6 h at 28°C under light and dark conditions. After incubation, the plates were washed with distilled water to remove non-adherent cells, and 25 µL of 1% w/v Crystal Violet was added to each well and incubated for 15 min at room temperature. After removing the excess dye by washing the plates with distilled water, 200 µL of 95% v/v ethanol was added to each well and then transferred to a 1.5 mL eppendorf tube and the volume was adjusted to 1 mL with distilled water. Bacterial adhesion was quantified by determining the absorbance at 540 nm.

*X. axonopodis* pv. citri adhesion to biotic surfaces was assayed on orange leaves. Bacteria were grown and processed as described previously, but in this case, 20 µL of each bacterial suspension was placed on the abaxial face of the leaves and incubated for 6 h at 28°C in a humidified chamber in light and dark conditions. Bacterial adhesion was analyzed by Crystal Violet staining of the leaves for 15 min at room temperature, and the unbound dye was removed by gentle washing with distilled water. Bacterial attachment was measured by digital image analysis of the luminosity density of the spots (luminosity/pixel area).

### Plant Material and Inoculation

*Citrus sinensis* cv. Valencia orange plants were kindly provided by Catalina Anderson and Gastón Alanis (INTA Concordia, Argentina). Plants were grown in a greenhouse with a photoperiod of 16 h light (150 µE.m^−2^.s^−1^) and 8 h dark at a temperature of 25°C and 80% humidity.

For plant inoculation, bacteria were cultured in SB broth to an OD_600_ of 1, and cultures were adjusted to 10^7 ^CFU/mL with 10 mM MgCl_2_. Bacterial suspensions were infiltrated into the abaxial leaf surface using a needleless syringe. MgCl_2_ was used as a control for non-infected leaves. For the dark condition, the infiltrated leaves were protected from light with cardboard envelopes. To study *in planta* bacterial growth, leaves were taken at different days after infiltration and 0.8 cm diameter leaf discs were obtained from the infiltrated zones. The discs were ground in 100 µL of 10 mM MgCl_2_ followed by serial dilution and plating onto SB agar plates. Colonies were counted after 48 h of inoculation at 28°C. To quantify tissue damage we analyzed the percentage of necrotic area per infiltrated leaf area by digital image analysis. The pixels of the respective areas were measured using the histogram function of the program AdobePhotoshop 7.0 and the following rate was calculated: 100 X (necrotic area pixels/total infiltrated area pixels).

### Statistical Analysis

The quantitative analyses were performed with at least three independents biological samples. In each case, the plotted data correspond to the mean of these independent determinations, with the corresponding standard error indicated by the error bars. Data were subjected to a multifactorial ANOVA and Tukey’s multiple comparison tests using Infostat software (Infostat 2006®, http/www.infostat.com.ar). The outcome of the statistical analysis was represented using different letters to indicate the existence of statistically significant differences between the data or identical letters to indicate the absence of these differences.

## Supporting Information

Figure S1 Cloning of the *lov* gene and expression of Xac-LOV protein. (A) Steps for cloning of the *X. axonopodis* pv. citri *lov* gene in a pET-28a (+) vector for expression in the *Escherichia coli* BL21 (DE3) Codon Plus-RIL. (B) Sodium dodecyl sulfate-polyacrylamide gel electrophoresis (SDS-PAGE) gel of the elution fractions obtained from the purification of the Xac-LOV protein with a Ni-NTA-agarose resin. MW: molecular weight standards; 1: supernatant before purification; 2 and 3: washing column fractions; 4–20: elution protein fractions. In all cases, 10 µL of the corresponding sample were loaded into each well. F1 and R1: *X. axonopodis* pv. citri *lov*-specific forward and reverse primers, respectively. MCS: multiple cloning sequence.
(TIF)

Figure S2 Construction of *X. axonopodis* pv. citri Δ*lov* and Δ*lov-*p*lov* strains. (A) Steps for the generation of *X. axonopodis* pv. citri Δ*lov* mutant strain using the suicide plasmid pKMobGII to replace the *X. axonopodis* pv. citri *lov* gene with a Sm/Sp-resistance cassette. (B) Construction of the plasmid carrying a copy of the *lov* gene (promoter and coding region) for transformation of the Δ*lov* strain to generate *X. axonopodis* pv. citri Δ*lov*-p*lov* strain. (Ci) PCR with *X. axonopodis* pv. citri *lov*-specific primers F1 and R1 using genomic DNA from *X. axonopodis* pv. citri strains as template. 1: WT; 2: Δ*lov*; 3: Δ*lov-plov*; 4: positive control (p*lov*); 5: negative control (water). (Cii) Western blot analysis using polyclonal anti-Xac-LOV antibodies. UFS (upstream flanking sequence); DFS (downstream flanking sequence).
(TIF)

Figure S3 Bacterial growth curves in liquid SB medium. Saturated cultures of *X. axonopodis* pv. citri WT, Δ*lov* and Δ*lov*-p*lov* strains were subcultured into fresh SB medium at 2% v/v inoculums. Bacterial growth curves were obtained considering the optical density at 600 nm (OD_600_) (A) and the colony forming units (CFU)/ml (B) as a function of time. Data are represented as the mean +/− standard error of three independent biological samples and different letters above the bars indicate significant differences between the corresponding data (p<0.01).
(TIF)

Figure S4 Flagellin synthesis in *X. axonopodis* pv. citri Δ*lov* and Δ*lov-*p*lov* strains. Western blot analysis using polyclonal anti-flagellin antibodies from protein extracts of bacteria obtained from the migration zones of swarming plates.
(TIF)

Figure S5 Twitching-like motility of *X. axonopodis* pv. citri strains in dark conditions. *X. axonopodis* pv. citri WT, Δ*lov* and Δ*lov*-p*lov* strains were stab-inoculated on SB-1% w/v agar plates and grown for two days at 28°C in the absence of light. To analyze the borders of the migration zones, the plates were observed under a magnifying glass (10X), prior (upper panels) and after (lower panels) staining with Coomassie Brilliant Blue R250.
(TIF)

Figure S6 Analysis of extracellular structures of *X. axonopodis* pv. citri strains. *X. axonopodis* pv. citri WT, Δ*lov* and Δ*lov*-p*lov* colonies were analyzed on SB-1.5% w/v agar plates supplemented with 40 µg/mL Congo red dye.
(TIF)

Figure S7 Hydrogen peroxide resistance of *X. axonopodis* pv. citri WT, Δ*lov* and Δ*lov*-p*lov* strains. Cells in the early exponential phase of growth were exposed to the indicated concentrations of hydrogen peroxide (H_2_O_2_) for 15 min. The number of colony forming units (CFU) was determined for each culture before and after the peroxide treatment by plating appropriate dilutions. The percentage of survival is defined as the number of CFU after treatment divided by the number of CFU prior to treatment × 100. Data are represented as the mean +/− standard error of three independent biological samples and different letters above the bars indicate significant differences between the corresponding data (p<0.01).
(TIF)

Figure S8 Expression of *X. axonopodis* pv. citri adhesin gene in XVM2 medium. (A) Amplified products of the *fhaB* gene by semiquantitative RT-PCR using RNA preparations from early exponential *X. axonopodis* pv. citri cultures grown in XVM2. As a control for constitutive bacterial expression a fragment of 16S rRNA was simultaneously amplified. (B) Expression profiles obtained by densitometric quantification of band intensities. Data are expressed as the mean +/− standard error of three independent samples. I.O.D: integrated optical density; a.u.: arbitrary units.
(TIF)

Supporting Information S1 (DOC)
