# Supplementary figures and images for: A LOV Protein Modulates the Physiological Attributes of Xanthomonas axonopodis pv. citri Relevant for Host Plant Colonization
Source: PLoS One. 2012 Jun 4;7(6):e38226. doi: 10.1371/journal.pone.0038226 (PMC3366940; doi:10.1371/journal.pone.0038226)

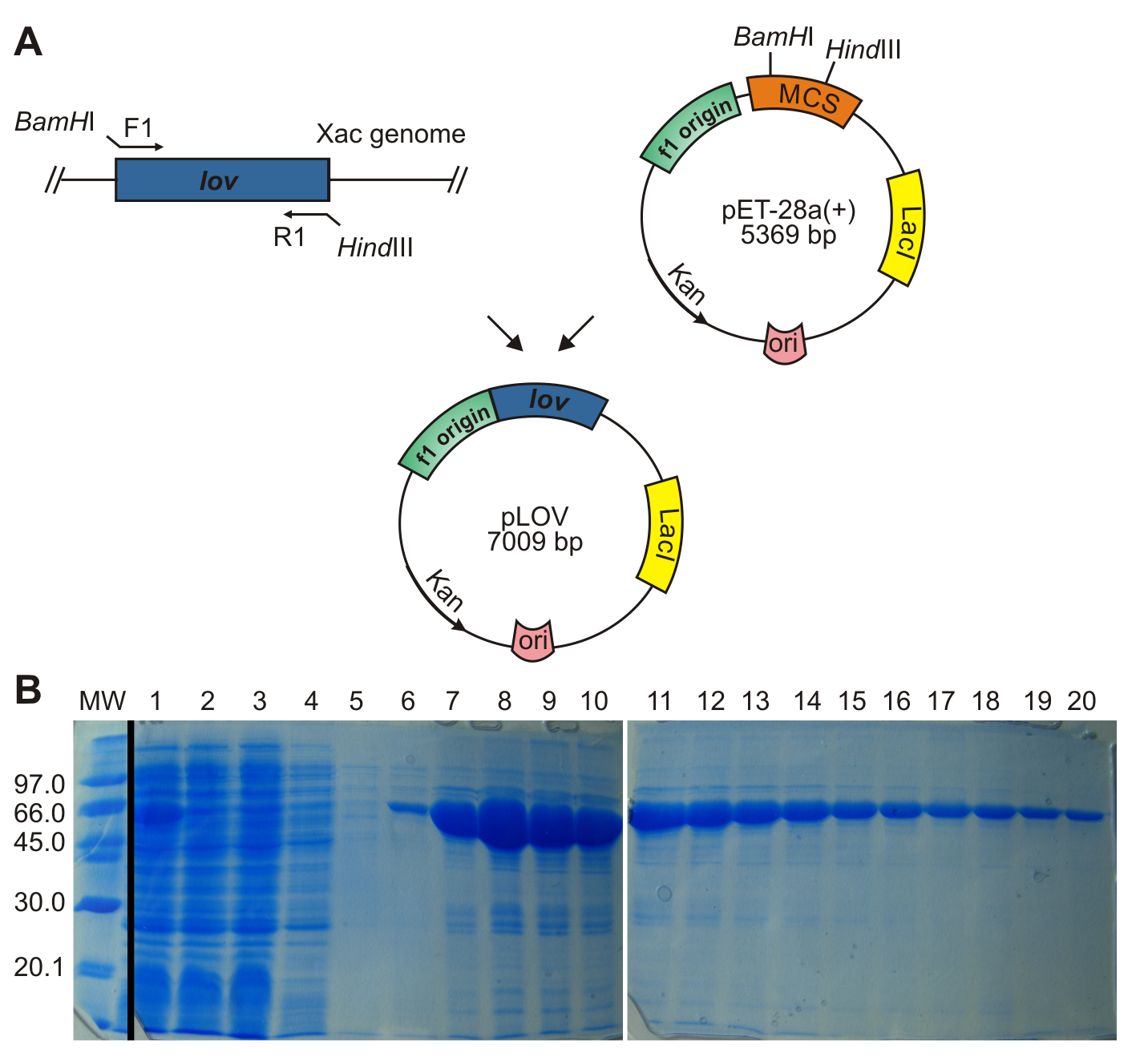

Supplement: Figure S1 — Cloning of the lov gene and expression of Xac-LOV protein. (A) Steps for cloning of the X. axonopodis pv. citri lov gene in a pET-28a (+) vector for expression in the Escherichia coli BL21 (DE3) Codon Plus-RIL. (B) Sodium dodecyl sulfate-polyacrylamide gel electrophoresis (SDS-PAGE) gel of the elution fractions obtained from the purification of the Xac-LOV protein with a Ni-NTA-agarose resin. MW: molecular weight standards; 1: supernatant before purification; 2 and 3: washing column fractions; 4–20: elution protein fractions. In all cases, 10 µL of the corresponding sample were loaded into each well. F1 and R1: X. axonopodis pv. citri lov-specific forward and reverse primers, respectively. MCS: multiple cloning sequence. (TIF) [file pone.0038226.s001.tif]

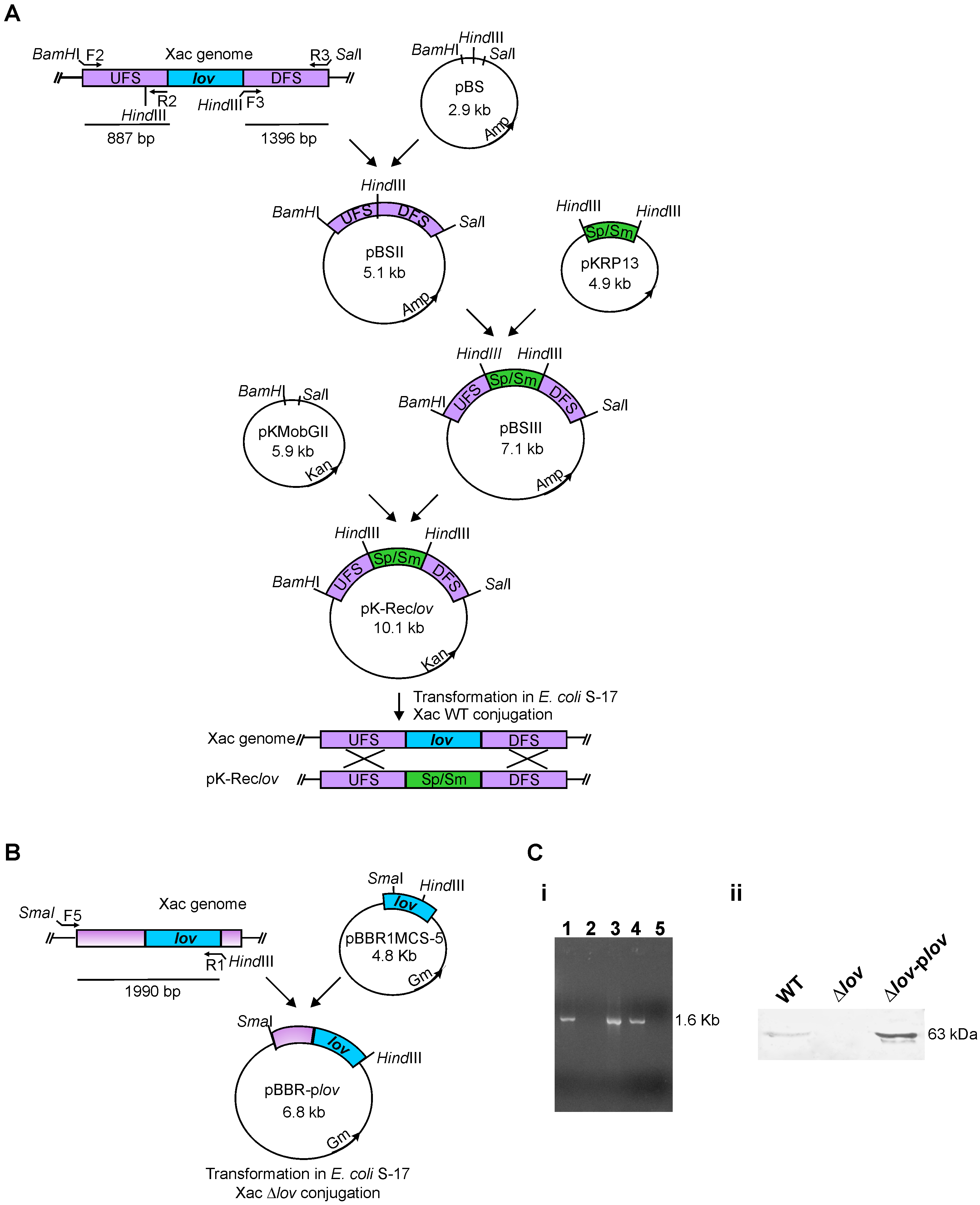

Supplement: Figure S2 — Construction of X. axonopodis pv. citri Δlov and Δlov-plov strains. (A) Steps for the generation of X. axonopodis pv. citri Δlov mutant strain using the suicide plasmid pKMobGII to replace the X. axonopodis pv. citri lov gene with a Sm/Sp-resistance cassette. (B) Construction of the plasmid carrying a copy of the lov gene (promoter and coding region) for transformation of the Δlov strain to generate X. axonopodis pv. citri Δlov-plov strain. (Ci) PCR with X. axonopodis pv. citri lov-specific primers F1 and R1 using genomic DNA from X. axonopodis pv. citri strains as template. 1: WT; 2: Δlov; 3: Δlov-plov; 4: positive control (plov); 5: negative control (water). (Cii) Western blot analysis using polyclonal anti-Xac-LOV antibodies. UFS (upstream flanking sequence); DFS (downstream flanking sequence). (TIF) [file pone.0038226.s002.tif]

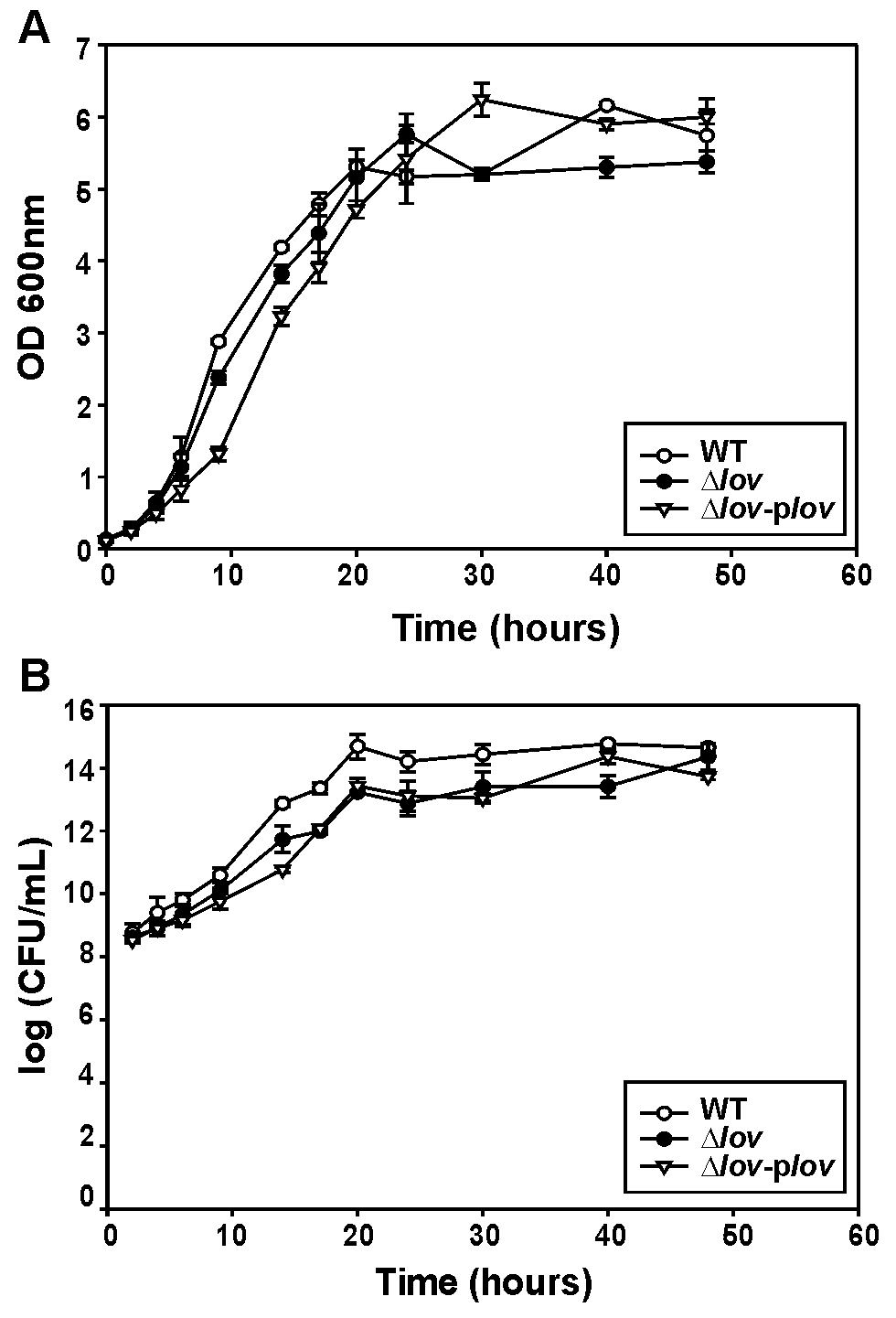

Supplement: Figure S3 — Bacterial growth curves in liquid SB medium. Saturated cultures of X. axonopodis pv. citri WT, Δlov and Δlov-plov strains were subcultured into fresh SB medium at 2% v/v inoculums. Bacterial growth curves were obtained considering the optical density at 600 nm (OD600) (A) and the colony forming units (CFU)/ml (B) as a function of time. Data are represented as the mean +/− standard error of three independent biological samples and different letters above the bars indicate significant differences between the corresponding data (p<0.01). (TIF) [file pone.0038226.s003.tif]

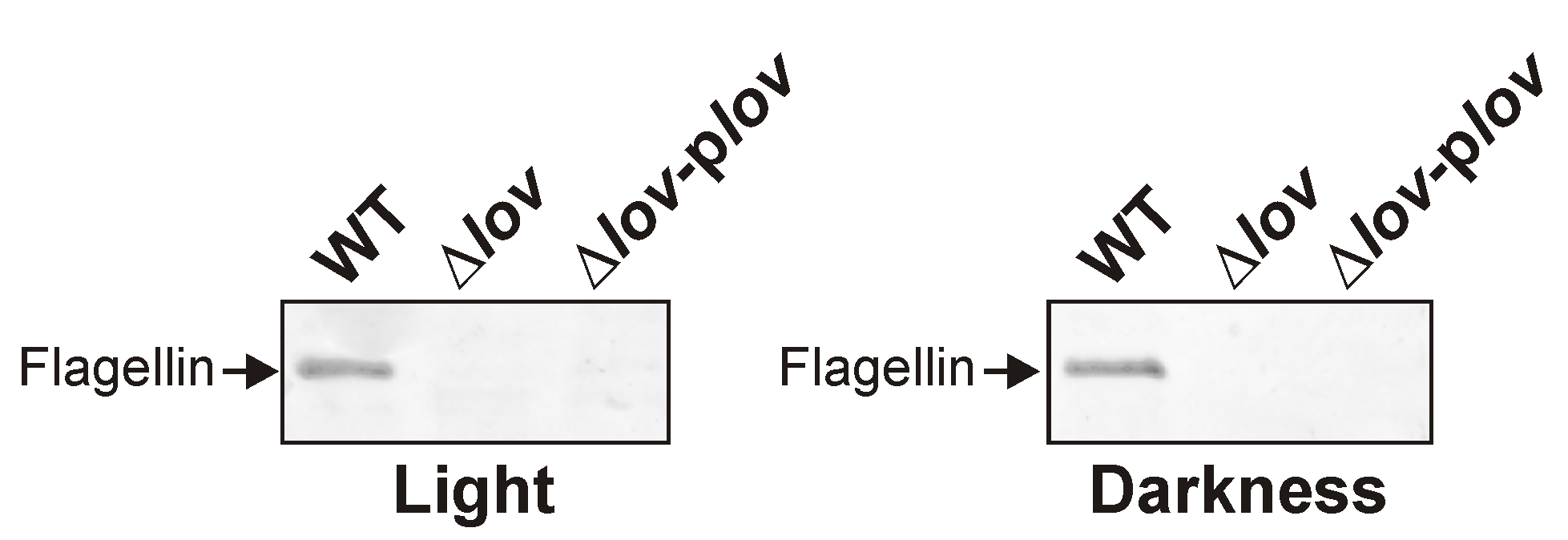

Supplement: Figure S4 — Flagellin synthesis in X. axonopodis pv. citri Δlov and Δlov-plov strains. Western blot analysis using polyclonal anti-flagellin antibodies from protein extracts of bacteria obtained from the migration zones of swarming plates. (TIF) [file pone.0038226.s004.tif]

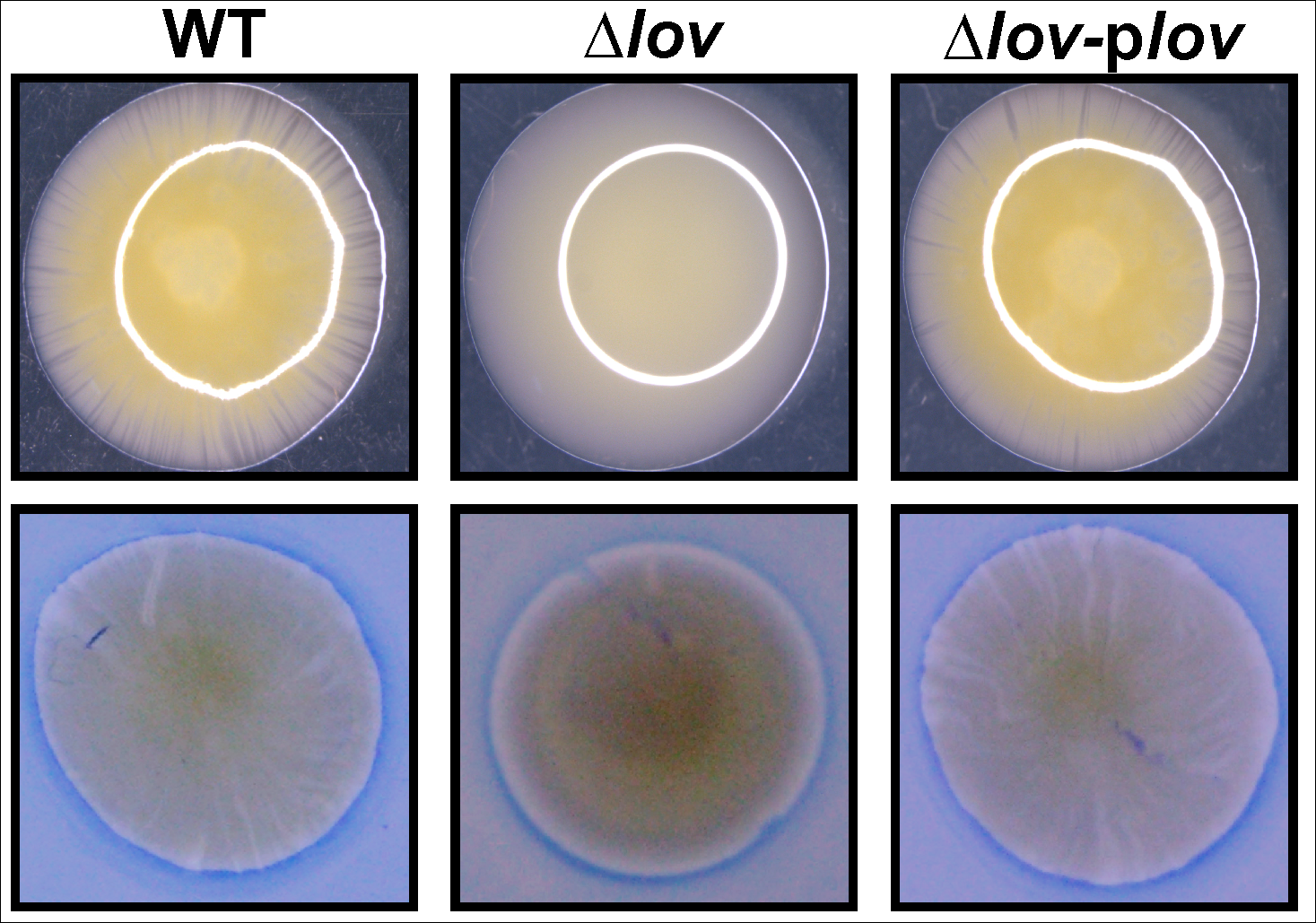

Supplement: Figure S5 — Twitching-like motility of X. axonopodis pv. citri strains in dark conditions. X. axonopodis pv. citri WT, Δlov and Δlov-plov strains were stab-inoculated on SB-1% w/v agar plates and grown for two days at 28°C in the absence of light. To analyze the borders of the migration zones, the plates were observed under a magnifying glass (10X), prior (upper panels) and after (lower panels) staining with Coomassie Brilliant Blue R250. (TIF) [file pone.0038226.s005.tif]

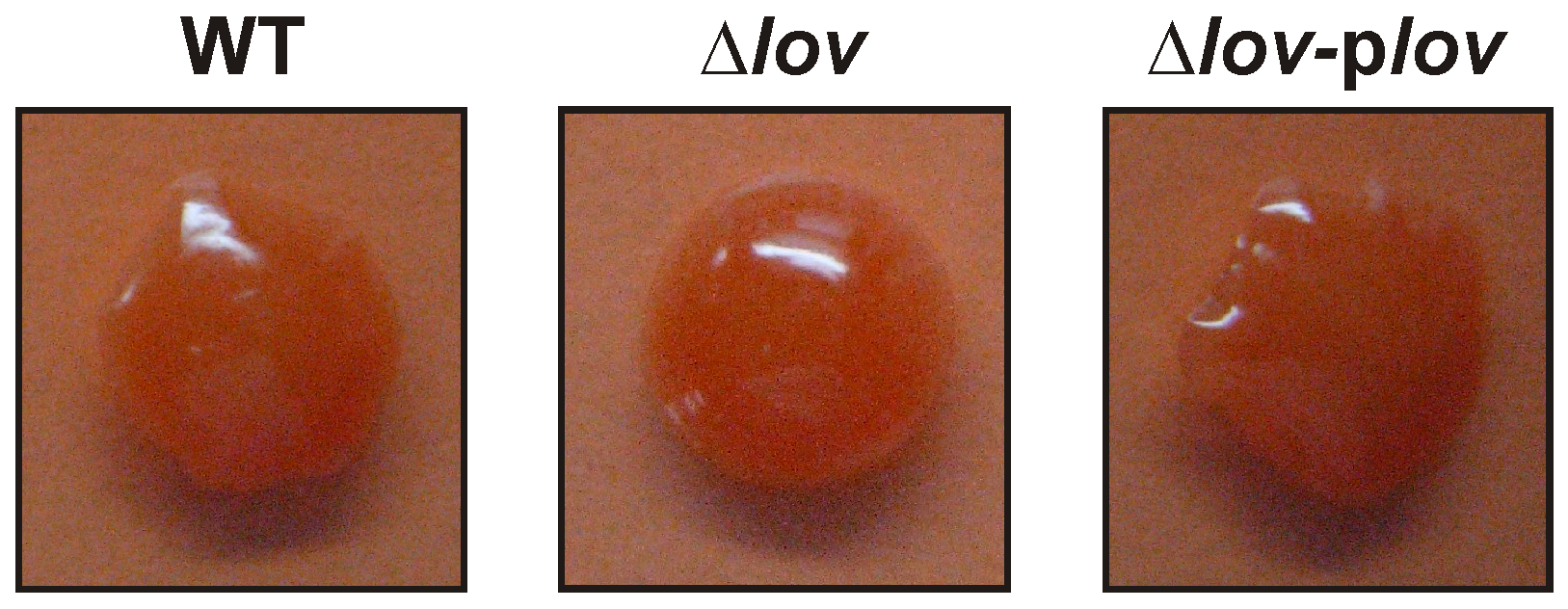

Supplement: Figure S6 — Analysis of extracellular structures of X. axonopodis pv. citri strains. X. axonopodis pv. citri WT, Δlov and Δlov-plov colonies were analyzed on SB-1.5% w/v agar plates supplemented with 40 µg/mL Congo red dye. (TIF) [file pone.0038226.s006.tif]

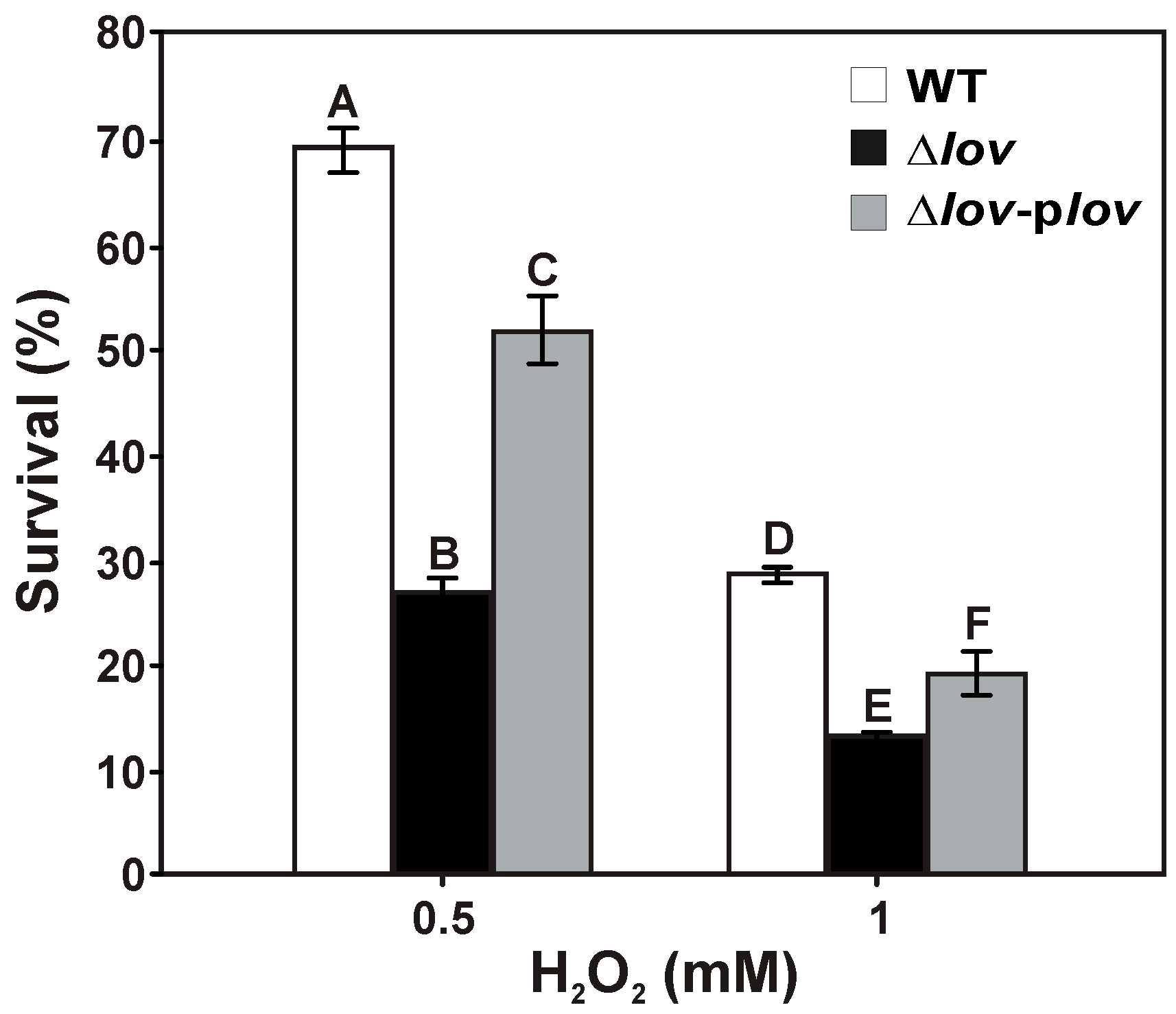

Supplement: Figure S7 — Hydrogen peroxide resistance of X. axonopodis pv. citri WT, Δlov and Δlov-plov strains. Cells in the early exponential phase of growth were exposed to the indicated concentrations of hydrogen peroxide (H2O2) for 15 min. The number of colony forming units (CFU) was determined for each culture before and after the peroxide treatment by plating appropriate dilutions. The percentage of survival is defined as the number of CFU after treatment divided by the number of CFU prior to treatment × 100. Data are represented as the mean +/− standard error of three independent biological samples and different letters above the bars indicate significant differences between the corresponding data (p<0.01). (TIF) [file pone.0038226.s007.tif]

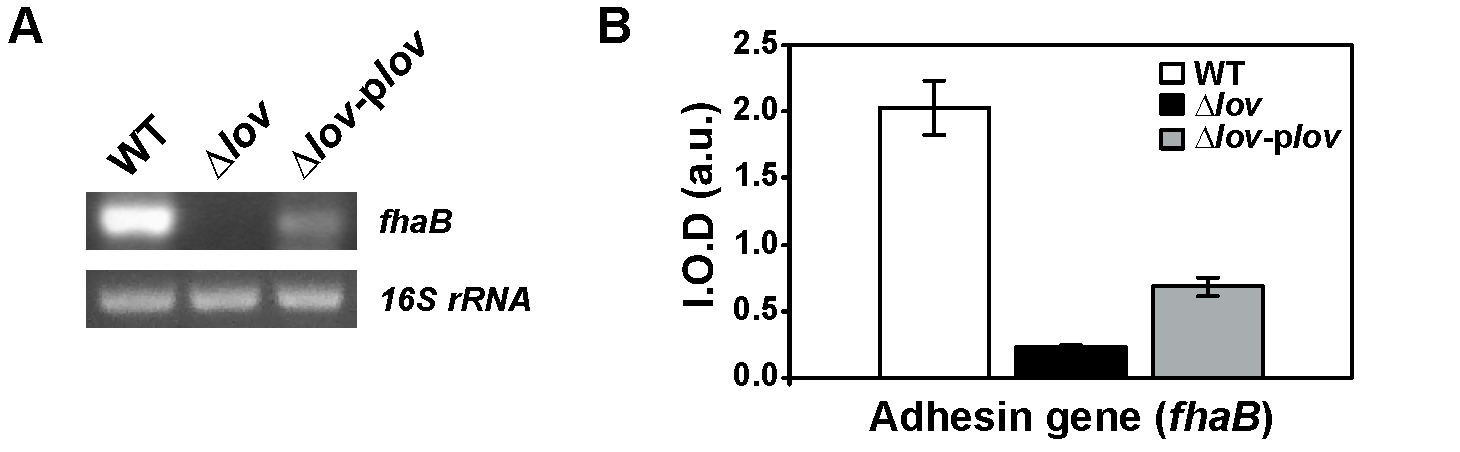

Supplement: Figure S8 — Expression of X. axonopodis pv. citri adhesin gene in XVM2 medium. (A) Amplified products of the fhaB gene by semiquantitative RT-PCR using RNA preparations from early exponential X. axonopodis pv. citri cultures grown in XVM2. As a control for constitutive bacterial expression a fragment of 16S rRNA was simultaneously amplified. (B) Expression profiles obtained by densitometric quantification of band intensities. Data are expressed as the mean +/− standard error of three independent samples. I.O.D: integrated optical density; a.u.: arbitrary units. (TIF) [file pone.0038226.s008.tif]
