# Supplementary material for: A LOV Protein Modulates the Physiological Attributes of Xanthomonas axonopodis pv. citri Relevant for Host Plant Colonization
Source: PLoS One. 2012 Jun 4;7(6):e38226. doi: 10.1371/journal.pone.0038226 (PMC3366940; doi:10.1371/journal.pone.0038226)
Supplement: Supporting Information S1 — (DOC) [file pone.0038226.s009.doc]

**A LOV Protein Modulates the Physiological Attributes of *Xanthomonas axonopodis* pv. citri Relevant for Host Plant Colonization**

Ivana Kraiselburd, Analía I. Alet, María Laura Tondo, Silvana Petrocelli, Lucas D. Daurelio, Jesica Monzón, Oscar A. Ruiz, Aba Losi and Elena G. Orellano

**SUPPORTING INFORMATION S1**

**METHODS AND RESULTS**

**Cloning and expression of the *Xanthomonas axonopodis* pv. citri *lov* gene**

We cloned and expressed the *Xanthomonas. axonopodis* pv. citri *lov* gene to obtain a purified recombinant Xac-LOV protein in *Escherichia coli*. To do this, standard microbiological and molecular genetics techniques were employed [1]. Total bacterial genomic DNA was isolated using the cetyltrimethylammonium bromide (CTAB) procedure [2]. Conjugation between *X. axonopodis* pv. citri and *E. coli* strains was performed as described by Simon *et al.* [3]. The steps for the cloning of the *X. axonopodis* pv. citri *lov* gene are shown in Figure S1A. The *X. axonopodis* pv. citri *lov* gene was amplified by PCR from *X. axonopodis* pv. citri wild-type (WT) genomic DNA with F1 (5’ CGGGATCCTTGAACGATCCCGGTCAC 3’) and R1 (5’ CCCAAGCTTTCAACCAACCCCGGTCGG 3’) as forward and reverse primers, respectively. These primers were designed to introduce *BamH*I and *Hind*III compatible ends, respectively. The 1.6 Kb product was cloned into the corresponding restriction sites of a pET 28a (+) vector, rendering the p*lov* plasmid in *E. coli* JM109. The presence of the *lov* gene in the kanamycin (Kan)-resistant clones was analyzed by colony-PCR using *X. axonopodis* pv. citri *lov*-specific primers. The recombinant Xac-LOV protein with a His-tail was expressed in the BL21 (DE3) Codon Plus-RIL (Stratagene) strain of *E. coli*. Induction was performed at 16 ºC with 0.5 mM IPTG for 18 h. Soluble protein was purified in batch with a Ni-NTA-agarose resin (Qiagen) according to the manufacturer’s instructions using a wash buffer containing 15 mM imidazole. Elution was performed with elution buffer containing 150 mM imidazole. Finally, elution fractions that presented a higher amount of Xac-LOV protein over contaminant proteins were pooled and dialyzed using a 12 kDa dialysis membrane. Protein was quantified as described by Sedmak and Grossberg [4]. Protein fractions were electrophoresed on 12% Sodium dodecyl sulfate-polyacrylamide gel electrophoresis (SDS-PAGE), and the gel was stained with Coomassie Brilliant Blue as described previously [1] (Figure S1B).

**Construction of the *X. axonopodis* pv. citri Δ*lov* mutant and complemented strains**

We constructed a deletion mutant in which the *X. axonopodis* pv. citri *lov* gene was replaced by a streptomycin/spectinomycin (Sm/Sp)-resistance cassette using the suicide plasmid pKMobGII [5]. The steps for the construction of this strain (Δ*lov*) are shown in Figure S2A. First, an 887-bp upstream flanking sequence (UFS) and a 1396-bp downstream flaking sequence (DFS) were generated by PCR from genomic DNA of *X. axonopodis* pv. citri using the primers F2 5’ CGGGATCCGGCAAGCAGCTACCCGAAC 3’ and R2 5’ CGCCAGATCACCCACGTC 3’ or F3 5’ CCCAAGCTTGAAGAGCGCATTCCAGAACG 3’ and R3 5’ ACGCGTCGACCGGTCCTAGGTCCTGCAATC 3’, respectively. These oligonucleotides were designed to generate *BamH*I and *Hind*III compatible ends in the UFS and *Hind*III and *Sal*I compatible ends in the DFS. The *Sal*I-*Hind*III-digested DFS was ligated to the corresponding restriction sites of pBluescriptSKII (+) (pBS) to generate the recombinant plasmid pBSI in *E. coli* JM109. The *BamH*I-*Hind*III-digested UFS fragment was ligated to the corresponding restrictions sites of pBSI to generate the plasmid pBSII in *E. coli* JM109. This last recombinant plasmid contains the sequences corresponding to the flanking regions (upstream and downstream) of the *X. axonopodis* pv. citri *lov* gene. The Sm/Sp-resistance cassette was released from the plasmid pKRP13 by digesting with *Hind*III and was subcloned into pBSII, generating the recombinant plasmid pBSIII in *E. coli* JM109 [6]. Finally, a 4.2 Kb DNA fragment consisting of the UFS fragment, the Sp/Sm-resistance cassette and the DFS fragment, was released from the pBSIII plasmid with *BamH*I and *Sal*I and cloned into the vector pKMobGII, generating a recombinant plasmid designated pK-Rec*lov*. *E. coli* S17-1 cells transformed with this plasmid were conjugated to the *X. axonopodis* pv. citri WT strain. The transconjugants were selected for Sm resistance and Km sensitivity.

For the construction of the Δ*lov-*p*lov* complemented strain, the *lov* gene with its promoter was amplified by PCR from *X. axonopodis* pv. citri genomic DNA with F4 (5’ TCCCCCGGGATACGCCTGCAACGTGAT 3’) and R1 as forward and reverse primers, respectively. These primers were designed to introduce *Sma*I and *Hind*III compatible ends, respectively. The 1.99 Kb product was cloned into the corresponding restrictions sites of a pBBR1MCS-5 [7] rendering pBBR-p*lov* plasmid in *E. coli* JM109. *E. coli* S17-1 cells transformed with this plasmid were conjugated to the *X. axonopodis* pv. citri Δ*lov* strain. The transconjugants were selected for Sm and Gentamycin (Gm) resistance (Figure S2B).

The absence of the *lov* gene in the Δ*lov* strain and its reinsertion in the complemented strain were verified by PCR using the F1 and R1 primers. PCR products were separated on 1% w/v agarose gels that were ethidium bromide-stained. The PCR conditions were as follows: 5 min at 95 ºC; 10 cycles of 1 min at 95 ºC, 1 min at 56 ºC and 1 min at 72 ºC; 20 cycles of 1 min at 95 ºC, 1 min at 65 ºC and 2 min at 72 ºC; and one final extension cycle of 5 min at 72 ºC (Figure S2Ci). We also evaluated the synthesis of Xac-LOV protein in *X. axonopodis* pv. citri strains by western blot analysis using polyclonal anti-Xac-LOV antibodies obtained in rabbit using the purified recombinant Xac-LOV protein as described by Houghton [8]. Bacteria were cultured in SB medium to the late exponential phase in light and dark conditions. Bacteria were harvested by centrifugation and resuspended in 500 μL PBS buffer. Protein extraction and western blot analysis were performed as described by Sambrook *et al.* [1] using identical amounts of each protein extract (Figure S2Cii). We observed an immunoreactive band for *X. axonopodis* pv. citri WT and complemented strain.

For the construction of the Δ*lov-*p*lov*’ complemented strain, the *lov* gene with its promoter was amplified by PCR from *X. axonopodis* pv. citri genomic DNA with F5 (5’ CCGCTCGAGGttgaacgatcccggtca 3’) and R1 as forward and reverse primers, respectively. These primers introduce *Xho*I and *Hind*III compatible ends, respectively. The 1.64 Kb product was cloned into the corresponding restrictions sites of a pBBR1MCS-2 [7] rendering pBBR-p*lov*2 plasmid in *E. coli* JM109. *E. coli* S17-1 cells transformed with this plasmid were conjugated to the *X. axonopodis* pv. citri Δ*lov* strain. The transconjugants were selected for Sm and Km resistance. The reinsertion and expression of the *lov* gene in the Δ*lov-*p*lov*’ strain was verified by PCR and western blot analysis as was described above.

**Growth curves in liquid medium**

The viability of the Δ*lov* and Δ*lov*-p*lov* strains was evaluated by growth curves in liquid SB medium. These were performed by subculturing overnight cultures of *X. axonopodis* pv. citri strains into fresh SB medium at 2% v/v inoculum. Aliquots of the cultures were taken at different times to measure optical density at 600 nm (OD_600_) and were plated on SB-1.5% w/v agar plates to evaluate colony-forming capacity. Plots of OD_600_ and Log colony forming units (CFU)/mL were obtained as a function of time. As shown in Figure S3, both strains presented growth curves in liquid media identical to that obtained for the WT, implicating that the deletion of the *lov* gene does not affect *X. axonopodis* pv. citri viability or growth rate. The Δ*lov*-p*lov*´ strain presented the same viability of the WT strain.

**Flagellin analysis by Western Blot**

We analyzed the synthesis of flagellin by the different *X. axonopodis* pv. citri. For the inmunodetection of flagellin protein, bacteria from the migration zones of swarming plates were collected and resuspended in 500 μL PBS buffer (8 g/L NaCl, 1.15 g/L Na_2_HPO_4_·7H_2_O, 0.2 g/L KH_2_PO_4_, pH =7.4). Protein extraction and western blot analysis were performed as described by Sambrook *et al.* [1], using identical amounts of each protein extract. Polyclonal anti-flagellin rabbit antibodies from *Serratia marcesens* were kindly provided by Dr. Eleonora García Véscovi. As shown in Figure S4, we could observe an immunoreactive band for *X. axonopodis* pv. citri WT in both growth conditions. However, the band corresponding to flagellin was hardly detected for the mutant and complemented strains.

**Twitching motility**

Twitching motility was analyzed as described in the Materials and Methods section. In this case, twitching plates were covered with an aluminum foil to generate the dark condition. As shown in Figure S5, we observed bacterial extensions irradiating from the migration zones generated by the WT and Δ*lov*-p*lov* strain, but not by the Δ*lov* strain, which showed smooth margins. This result is similar to the one obtained in light conditions (Figure 4).

**Analysis of extracellular structures by Congo red staining**

We analyzed the colonies developed by the WT, Δ*lov* and Δ*lov*-p*lov* strains of *X. axonopodis* pv. citri in SB-1.5 % w/v agar plates containing 40 μg/mL Congo red, a dye used for the detection of extracellular fibers such as the curly fibers observed in several pathogenic bacteria [9]. In spite of observing differences in the morphology of the colonies, no difference were observed in the interaction with the Congo red dye, implicating that the different *X. axonopodis* pv. citri strains have no modifications in this type of structures (Figure S6).

**Hydrogen peroxide resistance**

To investigate whether the deletion of the *X. axonopodis* pv. citri *lov* gene modifies its resistance to oxidative stress, we performed studies of bacterial survival in the presence of hydrogen peroxide. Survival experiments were performed by subculturing *X. axonopodis* pv. citri overnight cultures into fresh SB medium at 2 % v/v inoculum. After 6 h of growth (exponential phase), aliquots of the cultures were diluted and plated on SB-1.5 % w/v agar plates. Hydrogen peroxide was added to the cultures at final concentrations of 0.5 and 1 mM. After 15 min of exposure to the oxidant, samples were removed, washed once with fresh medium, serially diluted and plated on SB-1.5 % w/v agar plates. In all cases, the growth of liquid cultures was monitored spectrophotometrically by measuring OD_600_. Colonies were counted after two days of incubation at 28 ºC. The percentage of survival was defined as the number of CFU after treatment divided by the number of CFU prior to treatment × 100. As shown in Figure S7, in the early exponential phase, *X. axonopodis* pv. citri Δ*lov* was very sensitive to hydrogen peroxide treatment in a dose-concentration manner, diminishing its survival from 28 % to almost 10 % following addition of 0.5 mM and 1 mM hydrogen peroxide, respectively. In contrast, the WT strain presented a 70 % survival after the addition of 0.5 mM hydrogen peroxide, which was reduced to almost 28 % after treatment with 1 mM hydrogen peroxide. The Δ*lov*-p*lov* strain presented a survival level intermediate between *X. axonopodis* pv citri WT and Δ*lov* strains. This result shows a possible protective role of the Xac-LOV protein against oxidative stress.

**RNA extraction and semi-quantitative reverse transcription PCR (RT-PCR)**

For the analysis of adhesin expression total RNA of *X. axonopodis* pv. citri cells cultured in XVM2 medium at exponential phase was isolated using TRIzolH reagent (Invitrogen), according to the manufacturer’s instructions. After extraction, the RNA was treated with RNase-free DNase (Promega) and its integrity was checked by agarose gel electrophoresis. The semi-quantitative analysis of transcript levels of *fhaB* gene was carried out with a two-step RT-PCR approach using the gene-specific primers FhaBdown (5’ Catcgatgctcagctggttag 3’**)** and FhaBup (5’ ggaagcatgctcagtctggt 3’), which amplify a fragment of 280 bp of the *X. axonopodis* pv. citri *fhaB* gene. For cDNA synthesis, 1 μg of total RNA was added to a 20 μL reverse transcription reaction medium containing 4 μL M-MLV buffer (Promega), 0.5 mM dNTP mixture, 0.5 μg FhaBdown primer, 200 U M-MLV reverse transcriptase (Promega) and incubated for 60 min at 42 °C. Reverse transcription was terminated by incubating for 5 min at 94 °C. Control reactions, where RT was omitted, were done in parallel for all the samples to rule out the possibility of amplification from contaminating DNA. PCR reactions were carried out with 1 μL cDNA template under the following conditions: 40 cycles of 1 min at 94 °C, 1 min at 59 °C and 30 s at 72 °C with a final extension step at 72 °C for 5 min. The number of cycles to be used, avoiding reaching the plateau of the PCRs, was previously determined by taking samples at different number of cycles during the PCR amplification step and analyzing the products obtained by agarose gel electrophoresis. As a constitutive control, a 217-bp fragment of 16S rRNA was amplified using the same PCR conditions but with only 25 cycles of amplification due to the high abundance of 16S rRNA in total RNA extracts. RT-PCR products were resolved on 2 % w/v agarose gels, and densitometrically quantified using Gel-Pro Analyzer Software 3.1 (Media Cybernetics). We could observe that while the WT strain of *X. axonopodis* pv. citri presented a high level of expression of the *fhaB* gene, the expression of this gene was hardly detectable for the Δ*lov* strain. For the Δ*lov*-p*lov* strain the expression levels of *fhaB* gene were intermediate between the WT and Δ*lov* strain (Figure S8). Experiments were performed in triplicate with similar results.

REFERENCES

1. Sambrook J, Fritsch EF, Maniatis T (1989) Molecular cloning. a laboratory manual. Cold Spring Harbor Laboratory Press, Cold Spring Harbor, NY, USA.

2. Murray MG, Thompson WF (1980) Rapid isolation of high molecular weight plant DNA. Nucleic Acids Res 8: 4321-4325.

3. Simon R, Priefer U, Pühler A. (1983) A broad host range mobilization system for in vivo genetic engineering: transposon mutagenesis in Gram-negative bacteria. Bio/Technology 1: 784-791.

4. Sedmak JJ, Grossberg SE (1977) A rapid, sensitive, and versatile assay for protein using Coomassie brilliant blue G250. Anal Biochem 79: 544-552.

5. Katzen F, Becker A, Ielmini MV, Oddo CG, Ielpi L (1999) New mobilizable vectors suitable for gene replacement in gram-negative bacteria and their use in mapping of the 3' end of the Xanthomonas campestris pv. campestris gum operon. Appl Environ Microbiol 65: 278-282.

6. Reece KS, Phillips GJ (1995) New plasmids carrying antibiotic-resistance cassettes. Gene 165: 141-1427.

7. Kovach ME, Elzer PH, Hill DS, Robertson GT, Farris MA, et al. (1995) Four new derivatives of the broad-host-range cloning vector pBBR1MCS, carrying different antibiotic-resistance cassettes. Gene 166: 175-176.

8. Houghton AN (1989) Antibodies: A Laboratory Manual edited by Ed Harlo and David Lane, Cold Spring Harbor Laboratory, 1988. ISBN 0 87969 314 2. Immunology Today 10: 390.

9. Römling U, Bian Z, Hammar M, Sierralta WD, Normark S (1998) Curli fibers are highly conserved between Salmonella typhimurium and Escherichia coli with respect to operon structure and regulation. J Bacteriol 180: 722-731.
